# Cell wall remodeling and polarized light analysis reveal ecotype-specific strategies in *Salicornia europaea* L. with biotechnological applications

**DOI:** 10.1038/s41598-025-30480-w

**Published:** 2025-12-08

**Authors:** Stefany Cárdenas Pérez, Katarzyna Niedojadło, Michał Świdzinski, Aleksandra Orzoł, Janusz Strzelecki, Agnieszka Piernik, František Kačík, Jaroslav Ďurkovič

**Affiliations:** 1 Department of Geobotany and Landscape Planning, Faculty of Biological and Veterinary Sciences, Nicolaus Copernicus University in Toruń, Lwowska 1, 87100 Toruń, Poland; 2 Department of Cellular and Molecular Biology, Faculty of Biological and Veterinary Sciences, Nicolaus Copernicus University in Toruń, Lwowska 1, 87-100 Toruń, Poland; 3 Department of Biophysics, Institute of Physics, Faculty of Physics, Astronomy and Informatics, Nicolaus Copernicus University in Toruń, Grudziądzka 5, 87-100 Toruń, Poland; 4 Department of Chemistry and Chemical Technologies, Technical University in Zvolen, T.G. Masaryka 24, Zvolen, 96001 Slovakia; 5 Department of Phytology, Technical University in Zvolen, T.G. Masaryka 24, 96001 Zvolen, Slovakia

**Keywords:** Cell wall remodeling, AFM, Nanomechanics, Pectin, Cellulose-lignin, Polarized light, Salinity-compartmentalisation, Industrial applications, Biochemistry, Biotechnology, Plant sciences

## Abstract

*Salicornia europaea*, a salt-tolerant halophyte, exhibits dynamic cell wall remodeling under salinity stress, offering a valuable model for understanding biopolymer adaptation and sustainable biomass valorisation. This study investigates how increasing NaCl concentrations (0, 200, 400, and 1000 mM) alter the composition and mechanical behaviour of key cell wall polymers cellulose, pectin, and lignin in an inland *S. europaea* population. Using atomic force microscopy, fluorescence-based imaging, pectin immunolocalization and polarized light microscopy, we demonstrate that salinity drives tiered changes in polymer deposition and stiffness. Optimal salinity (200–400 mM) induced cell wall softening, linked to reduced cellulose deposition and increased pectin methylesterification, which facilitate turgor maintenance and expansion. Lignin composition shifted toward syringyl-rich polymers, promoting elasticity and enhancing apoplastic water flow. At extreme salinity (1000 mM), cell walls exhibited reduced flexibility and altered lignin monomer profiles, favoring *p*-hydroxyphenyl units as a cost-saving adaptation. These biochemical shifts were accompanied by a spatial reorganization of tissue birefringence. By mapping polymer-specific responses to salinity, we provide a mechanistic framework for optimizing cell wall composition in *S. europaea* to enhance functional value. Our findings support the targeted cultivation of halophytes for applications in functional foods, plant-based therapeutics, and more efficient biofuel feedstocks under saline agriculture.

**Supplementary Information:**

The online version contains supplementary material available at 10.1038/s41598-025-30480-w.

## Introduction

*Salicornia europaea* L., commonly known as glasswort, is a halophytic plant that thrives in saline environments such as coastal marshes and salt flats. Its ability to survive in extreme salinity conditions has made it a focal species for studies on plant adaptations to abiotic stress. Salinity is one of the most significant environmental factors limiting plant growth and productivity, as it induces osmotic stress, ion toxicity, and oxidative damage^1^. Plants like *S. europaea* have developed unique strategies to cope with these stressors, including changes to their cellular structure and biochemical pathways. The plant cell wall, as the first barrier between the plant and its environment, plays a critical role in these adaptive responses^2^. The primary cell wall of growing plant cells is a dynamic and adaptable structure that balances strength with flexibility^3^. It expands irreversibly during growth through tightly regulated mechanisms that can respond within minutes. Shifts in apoplastic pH activate wall-loosening proteins like expansins^4,5^, enabling cell wall remodeling under salinity. Recent in situ studies show finely regulated spatial and temporal variation in wall polysaccharides, even within single wall layers, revealing microscale heterogeneity crucial for localized stress adaptation^3^.

The composition and nanomechanical properties of the cell wall represent a critical, yet frequently overlooked, aspect of a plant’s adaptive response to stress^6^. The plant cell wall is a dynamic structure that maintains integrity, regulates growth, and resists stress. Its main components—cellulose, pectin, and lignin—drive its mechanical and functional properties^7^. Salinity induced modifications in these components significantly influence the plant’s ability to endure stress conditions and maintain structural stability^2^.

Cellulose, the most abundant polysaccharide in the plant cell wall, provides tensile strength by forming micro-fibrils embedded within a matrix of hemicellulose and pectin. Studies have shown that salinity stress can lead to alterations in cellulose rigidity, potentially affecting the structural integrity of the cell walls^8,9^. These changes may influence cell expansion and flexibility, impacting overall plant resilience.

Pectins, another major component of the primary cell wall, are complex polysaccharides that include homogalacturonans (HGs) as well as rhamnogalacturonans (RG-I and RG-II). Galacturonic acid (GalA) is the main sugar in the backbone of all three classes of pectins, and its carboxyl groups can be methyl-esterified. These methyl groups can hinder Ca²⁺-mediated ionic cross-linking, thereby influencing cell wall porosity, adhesion, and mechanical flexibility. Pectins are categorized based on their degree of methylation into high- methylesterified (HM-HG) and low- methylesterified pectins (LM-HG), both of which contribute to the cell wall’s dynamic properties. Salinity stress can alter the degree of pectin methylation, influencing the elasticity and plasticity of the cell wall, which in turn impacts the plant’s ability to withstand osmotic stress^10^.

Lignin is a complex aromatic polymer that strengthens secondary cell walls, enhancing structural support and stress tolerance. Under saline conditions, shifts in lignin composition—particularly in S, G, and H monomer ratios have been reported in several plant species and are considered part of adaptive mechanisms to maintain wall integrity^11^. Hydrophobic lignin not only strengthens cell walls but also contributes indirectly to mineral transport by forming apoplastic barriers that regulate water and solute pathways, while limiting water penetration and transpiration. This helps to maintain osmotic balance and membrane stability during high salt stress^12^. In *Salicornia bigelovii*, increasing salinity has been shown to reduce lignin content in stems and seed spikes^13^. The S/G and H/G ratios are key indicators of lignin composition, with S and G units forming the polymer backbone through labile β-O-4 linkages^14^. G-rich lignin has more C–C bonds via its reactive C-5 position, promoting condensation, whereas a higher S/G ratio favors longer, more linear chains and a more open matrix^15^. Elevated H content is linked to lower molecular weight, reduced cross-linking, and weaker structures. Although the functional role of H/G ratios under salinity remains underexplored, lignin heterogeneity supports the plausibility of H/G-driven structural modulation^11,16^.

Such compositional characteristics have direct implications for industrial applications: lignin with higher S/G ratio is more amenable to enzymatic hydrolysis, enhancing saccharification efficiency for bioethanol production^17–20^, while tailored lignin profiles can also improve the extraction of bioactive compounds for pharmaceutical use and enhance functional properties relevant to food formulations. In a previous study on a high salt-tolerant *S. europaea* population, an elevated S/G ratio was observed at optimal salinity (≤ 400 mM), while extreme salinity reduced S units, highlighting salinity’s impact on cell wall composition and its relevance for food, pharmaceutical, and biomass applications^2^.

Beyond cell wall polymer changes, polarized light microscopy (PLM) is a valuable tool for analyzing salt-induced birefringence in plant tissues. Here, PLM was used to visualize birefringence patterns linked to cell wall organization^21^. In the context of *S. europaea*, birefringence signals offer a non-destructive means to detect structural changes that may be influenced by saline environments. Although PLM does not directly detect salt crystals, it offers an indirect way to assess salt-induced structural organization at the tissue level. Combining PLM with techniques such as Raman microspectroscopy or cryo-SEM/EDS could further clarify the mechanisms behind these patterns, shedding light on *Salicornia*’s adaptation to extreme salinity.

*S. europaea* populations exhibit different performance under salinity, often linked to their geographical origin and local environmental conditions^22,23^. The *S. europaea* inland population from Ciechocinek, located in a natural brine region of Poland, represents an isolated population of particular interest due to its distinct physiological performance under salinity, differing from other reported populations, including the Inowrocław population located approximately 40 km from Ciechocinek, as well as from other studied German populations^2,24,25^. Investigating the underlying mechanisms of this particular population could provide broader insights into different plant adaptation strategies under saline conditions.

Halophytes respond to salinity through structural and biochemical adjustments, including cell wall remodeling of pectin, cellulose, and lignin, which supports mechanical stability and osmotic regulation across species^2,23,26^. However, little is known about how these responses vary among ecotypes, especially in inland *S. europaea* under different salinity regimes.

Previous studies have characterized a “typical” remodeling strategy marked by early wall softening and S/G shifts in Inowrocław Polish population^2^. Whether all populations follow this model or show ecotype-specific strategies remains unclear. Here, we examine the Ciechocinek population from Poland, which thrives under higher, stable salinity, to quantitatively assess its remodeling trajectory under increasing salt stress.

This study was aimed to examine how salinity impacts the composition of lignin, pectin, and cellulose in the cell walls of an isolated *S. europaea* inland population from Ciechocinek, how these changes correlate with the mechanical properties of cell walls particularly Young’s modulus (*E*) as a measure of stiffness and how intracellular salt accumulation patterns evolve under saline conditions, as visualized through polarized light microscopy. By analysing the impact of varying salinity levels on these structural components, this research sought to elucidate how cell wall modifications contribute to plant adaptation under salt stress. We hypothesize that although cell wall remodeling is a general adaptive mechanism in *S. europaea*, the Ciechocinek ecotype exhibits a distinct and quantifiable strategy, which can be differentiated from other populations. Specifically: 1. The Ciechocinek ecotype may display delayed wall softening under increasing salinity compared to the previous studied Inowrocław population. 2: The polymer composition (pectin, cellulose, and lignin) can reflect ecotype-specific quantitative shifts along the salinity gradient. 3. The S/G and H/G lignin ratios in Ciechocinek reflect a conservative remodeling strategy, contrasting with the early softening and compositional shifts observed in the Inowrocław population.

These ecotype-specific remodeling responses may ultimately be shaped by local environmental salinity regimes, providing a mechanistic basis for adaptive differentiation in *S. europaea* a hypothesis to be explored in future studies through integrated ecological and epigenetic approaches. In addition, beyond its ecological significance, *Salicornia*’s ability to thrive in extreme saline conditions makes it a promising candidate for applications in food, pharmaceuticals, and biofuel production. *S. europaea* is rich in bioactive compounds, including antioxidants, polyphenols, and essential minerals, which contribute to its potential as a functional food ingredient^27,28^. Additionally, by knowing its lignocellulosic biomass composition, it can serve as a sustainable source for bioethanol and biodiesel production, addressing the growing demand for renewable energy sources^28,29^. Furthermore, the plant’s salt-tolerant properties offer potential in pharmaceutical research, particularly in the development of stress-resistant crops and halophyte-derived medicinal compounds^30,31^. Importantly, histochemical approaches provide powerful tools to enhance these applications. They allow precise localization and quantification of key metabolites (e.g., lignin, cellulose, pectins), supporting their targeted production or extraction, while also revealing structural changes linked to improved stress tolerance, biomass quality, and crop performance. Therefore, the present multidimensional study contributes to a deeper understanding of the role of cell wall remodeling in plant adaptation to salt stress, with implications for these diverse biotechnological applications.

## Materials and methods

### Plant growth conditions and salinity treatments

*S. europaea* seeds were collected from the inland salt marsh of Ciechocinek (Cie), Poland (52°53′N, 18°47′E). Located in the nature reserve protecting inland salt marshes, which was established in 1964^32^. Due to the historical drainage of the Vistula Valley, halophytic vegetation is now restricted to a ditch system crossing the reserve. It is not regularly supported by brine from the graduation towers nearby, but the area is characterized by high soil salinity (~ 100 dS m^− 1^ or ~ 1000 mM NaCl)^33^.

The seeds were germinated and cultivated following previously reported protocols^23,34^, with slight modifications in salinity treatments at 0, 200, 400, and 1000 mM NaCl. Germination was conducted in Petri dishes (Ø 7 cm) with filter paper and 5 ml of distilled water in a growth chamber. Once germinated, seedlings were transferred to individual pots (5.3 cm height, 5.5 cm diameter, 125 cm^3^) containing − 1:1 vermiculite-sand substrate, with one plant per pot and 12 seedlings per treatment. Before planting, each set of 12 pots was placed in undrained trays and saturated with 500 ml of the respective saline solution (0, 200, 400, or 1000 mM NaCl) to the full capacity. Growth chamber conditions were maintained at 25/20°C (day/night), a photon flux density of 1000 mmol m⁻² s⁻¹, relative humidity of 50–60%, and a 16/8 h (light/dark) photoperiod. For the first 21 days, seedlings were irrigated with distilled water (250 ml) via tray watering, followed by a 30-day period of irrigation with Hoagland’s solution every two days to ensure consistent salinity and nutrient supply. A total of 48 plants (12 per treatment) were cultivated. After 60 days of salinity treatments, nanomechanical properties, structure-function analysis, and microscopy observations were performed.

Salinity treatments of 0, 200, 400, and 1000 mM NaCl were chosen to span a gradient from below-optimal (0 Mm) to extreme stress conditions. Levels of 200–400 mM are routinely used in *S. europaea* studies to mimic optimal–high salinity^2,22–24^ while maintaining vegetative growth, whereas 1000 mM was included as a supra-optimal treatment to probe the upper limits of cell-wall plasticity and nanomechanical adjustment, despite not supporting full life-cycle completion.

The collection and handling of plant material complied with institutional, national, and international guidelines, including the IUCN Policy Statement on Research Involving Species at Risk of Extinction and the Convention on the Trade in Endangered Species of Wild Fauna and Flora. A voucher specimen has been deposited in the publicly available herbarium of Nicolaus Copernicus University in Toruń (Index Herbarium code TRN). Although the deposition number is not available, the species was formally identified by Dr. hab. Agnieszka Piernik, Prof. NCU, and permission to work with the seeds was granted by the Regional Director of Environmental Protection in Bydgoszcz (WOP.6400.12.2020.JC).

### Tissue preparation for microscopy observations

For microscopy analyses including cellulose and lignin localization, as well as intracellular salt distribution patterns visualized via polarized light fresh tissue sections were obtained from the fleshy shoot segments of *S. europaea* plants subjected to 0, 200, 400, and 1000 mM NaCl treatments. Transverse Sections (150 μm thick) were cut using a Leica VT1000S vibratome. The most intact and sharply defined slices were selected for further observation. For each treatment condition, tissue samples from three individual plants were analyzed.

### Nanoindentation-based cell wall mechanical evaluation using AFM

The nanomechanical properties of *S. europaea* cell walls were assessed in plants grown under 0, 200, 400, and 1000 mM NaCl treatments. Stem cross-sections (~ 0.5 mm thick) were prepared for nanoindentation following the protocol described by Cárdenas-Pérez et al., ^2^. All measurements were performed using a Bruker Bioscope 2 atomic force microscope (AFM).

Tipless TL-FM cantilevers (Nanosensors) were modified into colloidal probes by attaching 25 μm polystyrene beads (microParticles GmbH) with epoxy adhesive (UHU Endfest), following the standard procedure described by Bruker^35^. Prior to each measurement session, cantilevers were calibrated in liquid using the thermal tune method^36^. Deflection sensitivity (def.s.) was determined on a rigid gold-coated glass substrate, assuming zero indentation (*δ* = 0), with def.s. calculated in nm/V from the equation z = δ + def.s. The resulting def.s. was approximately 75 nm/V, and the spring constant (*k*) was determined to be within 1–4 N/m range.

Nanoindentation was performed on water-storing parenchyma cells, with precise positioning of the AFM cantilever achieved using an integrated optical microscope and motorized stage. Depending on surface topography, more than five regions per sample were selected for indentation. At each location, over 30 force curves were collected within a 10 μm radius.

The *E* was calculated by fitting the Hertz model (Eq. 1) to the indentation slope of the obtained retrace curves.

1 $$\:F=\frac{4}{3}\frac{E}{\left(1-{\nu\:}^{2}\right)}\sqrt{R}{\delta\:}^{3/2}$$

where *F* is force, *E* is Young’s modulus, *R* is the indenter tip radius (12.5 μm), *δ* is the indentation depth (nm), and *ν* is Poisson’s ratio. A relative force trigger exceeding 100 nN was applied during indentation. For elastic materials such as plant cells, Poisson’s ratio typically ranges from 0.3 to 0.5^37^. In this study, a value of ν = 0.5 was used for all calculations of *E*. Model fitting to the experimental force–indentation curves was performed using NanoScope Analysis software 3.0 (Bruker, USA), following the methodology outlined by Cárdenas-Pérez et al.,^2,38^. The quality of the fit was assessed via the coefficient of determination (R²), and any curves with R² < 0.8 were excluded from further analysis. Cell wall nanoindentation experiments were performed on three cross sections per treatment, recording over 1000 force–indentation curves across multiple tissue regions.

### Sampling and preparation of stem sections

Three plants per treatment were used for selected microscopy analyses. From each plant, three stem cross-sections were obtained from the middle portion of the main shoot, ensuring anatomical consistency along the stem axis. The same sampling scheme was applied across all imaging techniques, including polarized light microscopy, fluorescence microscopy, and histochemical staining, resulting in a total of nine cross-sections per treatment.

### Cellulose detection in *S. europaea* tissues via fluorescence microscopy

The calcofluor white staining protocol was used for detecting cellulose in plant cell walls due to its high specificity for β-linked glucans^39^. Fresh plant cross-sections were transferred to distilled water to maintain tissue integrity. Samples were directly immersed in an aqueous solution of Calcofluor White (0.01–0.1% w/v), a fluorescent dye that selectively binds to cellulose and other β-glucans^40^. The samples were incubated for 10–30 min at room temperature to allow the dye to penetrate the cell walls, ensuring uniform staining. Following incubation, the stained sections were washed for 1 min with distillated water and carefully transferred onto a clean glass slide and mounted with a coverslip to prevent drying and optical artifacts. Visualization was performed using a fluorescence microscope equipped with ultraviolet (UV) illumination (excitation at ~ 365 nm), under which cellulose-rich regions of the cell wall fluoresce bright, facilitating structural and compositional analyses^41^. The UV filter used was designed with a broad emission window that extends into the blue-green region. This method provides a rapid and reliable approach for cellulose detection in *Salicornia* plant tissues, aiding studies of cell wall architecture and modifications.

### Quantitative image analysis of cellulose fluorescence

To quantify the fluorescence intensity of cellulose-stained regions, ImageJ (v1.47) was used to analyze microscopy images. Fluorescence images were captured under consistent exposure settings and saved in TIFF format to avoid compression artifacts. The images were then processed in ImageJ by converting them to 8-bit grayscale and applying background subtraction to enhance signal clarity. A thresholding Otsu method^42^ was used to segment cellulose-rich areas, ensuring only relevant fluorescence was analyzed. The analyze-particles function measured the area and fluorescence intensity of the stained regions, with parameters set to exclude artifacts. Data were normalized against background fluorescence, and statistical analyses were performed to compare cellulose quantification across samples. To assess differences in cellulose distribution within the water cortical/parenchyma tissue, three radial regions of interest (ROI) were delineated: R1, positioned closest to the vascular bundle; R2, representing the intermediate parenchyma; and R3, situated near the cortical boundary.This approach enables both qualitative and quantitative evaluation of cellulose distribution in *Salicornia* plant tissues, providing valuable insights into cell wall composition and modifications.

### High and low- methylesterified pectin immunolocalization

Cross-sections of *S. europaea* tissue were embedded in BMM resin (butyl methacrylate, methyl methacrylate, 0.5% benzoyl ethyl ether, 10 mM DTT, Merck) following the procedure by Niedojadło et al.,^43^. Samples were cut into 1.5 μm semi-thin sections using a Leica UCT ultramicrotome and mounted on polysine slides (Thermo Fisher Scientific). Resin was removed with two acetone washes, followed by rinsing with distilled water and PBS (pH 7.2).

To prevent non-specific binding, sections were first incubated in 2% bovine serine albumin (BSA, Merck) in phosphate buffered saline (PBS) for 30 min at room temperature. Following this blocking step, they were incubated overnight at 4 °C with rat monoclonal anti-pectin antibodies (Plant Probes) either JIM7 (recognizing partially methylesterified homogalacturonan) or LM19 (recognizing partially and fully de-esterified homogalacturonan) diluted 1:50 in 0.2% BSA/PBS pH 7.2. After thorough washes with PBS pH 7.2, an AlexaFluor 488-conjugated goat anti-rat secondary antibody (Thermo Fisher Scientific) 1:1000 in 0.2% BSA/PBS pH 7.2 was applied for 1 h at 37 °C. The sections were washed, air-dried, and mounted using ProLong™ Gold Antifade Reagent (Thermo Fisher Scientific). Negative controls, prepared by omitting the primary antibody, confirmed the specificity of the labeling.

Fluorescence imaging was performed using an Olympus BX50 microscope with a 100x UPlanFI oil immersion lens and narrow band filters (U-MNU, U-MNG). Images were captured with an Olympus XC50 digital camera and analyzed using CellB software (Olympus Soft Imaging Solutions, Germany). For pectin immunolocalization, fluorescence intensity was quantified from the cortical/parenchymatic ROI, excluding vascular bundles.

### Fluorescence quantification of pectin immunolocalization

Quantitative fluorescence analysis was performed under standardized conditions, ensuring consistency in temperature, incubation times, and antibody concentrations. Image acquisition was carried out at 100x magnification using fixed exposure settings. Fluorescence intensity was measured in the cortical/parenchyma ROI across five semi-thin sections, and image processing was conducted using ImageJ (v1.47). Threshold levels were defined based on the autofluorescence of other metabolites observed in negative control samples. Signal intensity was expressed in arbitrary units (a.u.) as the mean fluorescence intensity per µm², following the methodology of Niedojadło et al.,^43^. Fluorescence observation was performed using three plants per treatment.

### Microscopic visualization of lignin and determination of lignin monomer composition

Lignin visualization in cell walls was carried out following the procedure by^44^. Plant tissues were stained with 0.02% toluidine blue O for 5 min, then rinsed five times with distilled water to remove excess dye. Stained images from the cortical/parenchyma ROI were analyzed using ImageJ. Images were converted to 8-bit and subjected to color thresholding to selectively binarize the bluish-green hue corresponding to lignified cell walls, while excluding the reddish-purple background. This approach enhanced the detection of lignin-positive regions that may not be visually distinguishable in raw images, allowing consistent quantification of lignified area across treatments. Lignin content was normalized to background intensity, and statistical analyses were conducted to compare samples. Toluidine blue O -based binarization was used as a semi-quantitative proxy, that it is sensitive to intensity shifts rather than specific chemical signatures, and future work will include more specific lignin staining or chemical mapping techniques (e.g., Raman spectroscopy, or FTIR imaging) to confirm these patterns.

Furthermore, analysis of nitrobenzene oxidation (NBO) products was performed on extracted stem biomass. Water- and ethanol-soluble extractives were removed using the standard NREL protocol^45^. Alkaline NBO followed a modified method by Kačíková et al.,^46^, in which 200 mg of extracted biomass was reacted with 5 ml of 2 M NaOH and 0.4 ml nitrobenzene at 180 °C for 2 h in sealed stainless steel vessels. After rapid cooling, excess nitrobenzene was extracted twice with dichloromethane. The aqueous phase was acidified with 2 M HCl (pH ~ 2.5) and extracted three times with dichloromethane. The combined organic phase was dried over anhydrous sodium sulfate, evaporated under nitrogen, and dissolved in methanol. NBO products were analyzed via HPLC (Agilent 1200, Agilent Technologies) using a diode array detector (240 nm) and a Kinetex C18 column (2.6 μm, 150 × 4.6 mm; Phenomenex). The mobile phase, water: methanol: acetonitrile: propionic acid (88:4:8:0.1), was run at 1 ml min^– 1^ at 35 °C. External standards from Sigma-Aldrich were used for identification and quantification. The S: G:H ratio in lignin was calculated as: S:G: H = (syringaldehyde + syringic acid) : (vanillin + vanillic acid) : (*p*-hydroxybenzaldehyde + *p*-hydroxybenzoic acid). These ratios describe the relative abundance of syringyl (S), guaiacyl (G), and *p*-hydroxyphenyl (H) monomers, which determine lignin’s structural properties and reactivity. Higher S/G ratios reflect a more linear, less condensed lignin enriched in β-O-4 linkages, whereas higher H/G ratios indicate enrichment in *p*-hydroxyphenyl units typically associated with stress conditions and lower biosynthetic cost^11,16,47^. Analyses were performed using four plants per treatment to confirm reproducibility.

### Polarized light microscopy of salt-induced birefringence in *S. europaea* tissue

Transverse fresh plant sections of *S. europaea* obtained from vibratome were transferred to distilled water to maintain tissue integrity. Sections were mounted in distilled water to minimize evaporation and maintain tissue hydration, thereby preserving the native optical characteristics of the samples, including any salt-induced structural features present in the apoplast. Observations were conducted using a PLM equipped with crossed polarizers and a rotatable stage. Birefringence patterns were analyzed by rotating the sample to detect anisotropic structures, with particular attention to intercellular spaces where salt accumulation is expected. The use of a full-wave plate (λ-plate, 530 nm) enhanced contrast, facilitating the differentiation between crystalline salt deposits and native cell wall structures. Bright birefringence along cell walls in the cortical/parenchyma ROI indicated the presence of salt-induced birefringent structures, likely influenced by tissue hydration and potential phase transitions, rather than direct visualization of crystalline salt alone.This methodological approach was developed based on previous studies reporting the importance of both intracellular and extracellular salt compartmentalization in halophytes^48,49^. For quantification, birefringent regions were identified through a the cortical/parenchyma brightness threshold in ImageJ by converting images to 8-bit grayscale and applying a threshold to isolate bright areas. The analyze-particles function was used to measure the area and intensity of birefringent regions, with background noise excluded. Analysis were performed using three plants per treatment. Data were normalized against un-brightened regions, and statistical analyses such as ANOVA were used to compare birefringence levels across samples.

### Statistical analysis

The salinity effects on nanomechanical functional plant adaptations, cellulose, pectin, lignin and salt polarized patterns quantification along the NaCl treatments were tested by one-way analysis of variance, Holm-Sidak method was used for comparison of the means through SigmaPlot v.14.0^50^. While for lignin monomer composition Duncan’s multiple range test was used for comparison of the means. Principal Component Analysis was developed in XLSTAT, 2023 v.1.4 Basic^51^ to assess the multivariate relationships among cell wall-related traits, including nanomechanical stiffness *E*, lignin monomer ratios, pectin, cellulose composition, FW and polarized brigthness -area and -intensity in tissue patterns across salinity treatments in the *S. europaea* population from Ciechocinek.

## Results

### Structural and functional adaptations of *S. europaea* under saline conditions

**Fig. 1:**
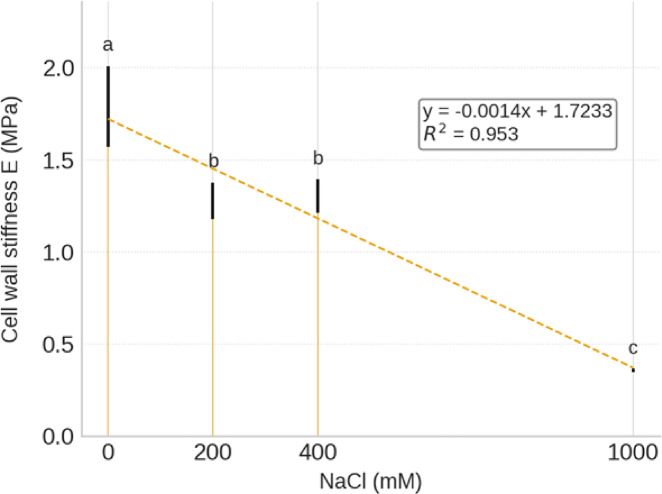
Effect of salinity on cell wall stiffness (Young’s modulus, *E*) in the water-storage parenchyma of *S. europaea*. Plants were subjected to increasing NaCl concentrations (0, 200, 400, and 1000 mM). Bars represent mean ± SE (*n* = 3). Different letters indicate statistically significant differences between treatments (*p* < 0.05). A linear regression (y = − 0.0014x + 1.723, R² = 0.95) describes the negative relationship between salinity and cell wall stiffness.

Cell wall stiffness (*E*) in the water-storage parenchyma of *S. europaea* was quantified across increasing NaCl concentrations (0, 200, 400, and 1000 mM). As shown in Fig. 1, a progressive reduction in stiffness was observed with increasing salinity. The highest *E* value (1.79 ± 0.22 MPa) was recorded under non-saline conditions (0 mM NaCl), while the lowest stiffness (0.36 ± 0.008 MPa) was measured at 1000 mM NaCl. Intermediate salinity levels (200 and 400 mM NaCl) induced a moderate decrease in stiffness (1.28 ± 0.10 and 1.30 ± 0.09 MPa, respectively). A linear regression analysis indicated a strong negative correlation between NaCl concentration and cell wall stiffness (R² = 0.95, *p* < 0.05), highlighting a clear trend of cell wall softening under saline stress.

Given these initial findings and the differences between the salinity gradient, we then examined the biopolymer characteristics and salt birefringent regions.

### Cellulose identification and quantification through fluorescence microscopy

**Fig. 2:**
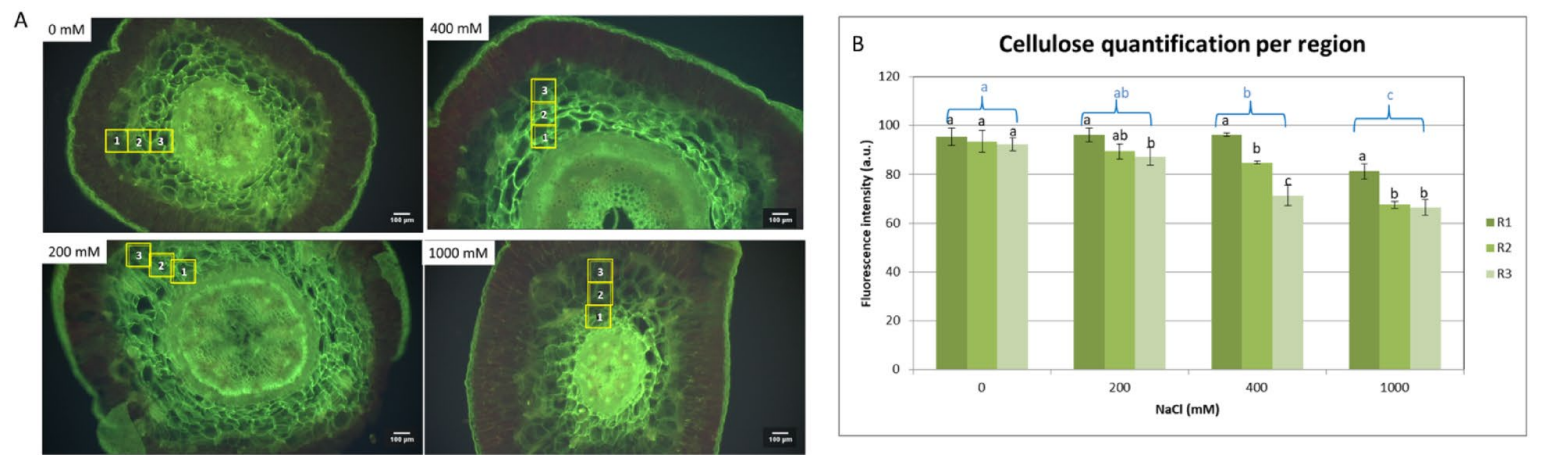
Cellulose fluorescence quantification across three tissue regions of *S. europaea* stem under NaCl treatments. (**A**) Representative fluorescence microscopy images of cross-sections from plants exposed to 0, 200, 400, and 1000 mM NaCl. Yellow boxes indicate the analyzed cortical/parenchyma ROIs: R1 – inner water parenchyma (adjacent to the vascular cylinder), R2 – intermediate water parenchyma, and R3 – outer water parenchyma (proximal to the cortex/epidermis). Scale bars: 100 μm. (**B**) Fluorescence intensity (a.u.) corresponding to cellulose quantified across regions. Data represent mean ± SD (*n* = 3 plants per treatment). Different letters indicate statistically significant differences between regions and treatments (*p* < 0.05).

Figure 2 shows quantitative and visual evidence of NaCl-induced changes in cellulose distribution across different tissue regions. Figure 2A presents fluorescence microscopy images of cross-sections from samples treated with 0, 200, 400, and 1000 mM NaCl. The green fluorescence signal corresponds to cellulose, with three distinct regions (R1 the inner zone adjacent to the vascular bundle; R2 the intermediate parenchyma layer; and R3 the outer zone located near the cortex) marked by yellow boxes. At 0 mM NaCl, fluorescence intensity is uniformly distributed across the examined regions. At 200 mM NaCl, fluorescence remains relatively stable across the tissue and cells appear more turgid. At 400 mM NaCl, fluorescence intensity decreases, particularly in R2 and R3, whereas in R1, cell turgidity and cell wall structure are maintained. At the highest NaCl concentration (1000 mM), fluorescence intensity is markedly reduced in comparison to lower saline treatments, all examined regions, with R2 and R3 showing the most pronounced decrease. Figure 2B quantifies fluorescence intensity across the three regions (R1, R2, and R3) along the salinity gradient. Through quantitative analysis we were able to find a significant reduction in cellulose fluorescence at 400 mM NaCl, observed in R2 and R3 compared to R1, as indicated by different statistical groupings letters. Quantitative analysis revealed that in 1000 mM NaCl, fluorescence intensity is further reduced across all examined regions, with R2 and R3 showing significantly lower values than R1. The statistical differences (*p* < 0.05) are denoted by different letters, highlighting the progressive cellulose modification under increasing NaCl concentrations.

### Confocal microscopy-based immunolocalization of high- and low-methylesterified pectins

**Fig. 3:**
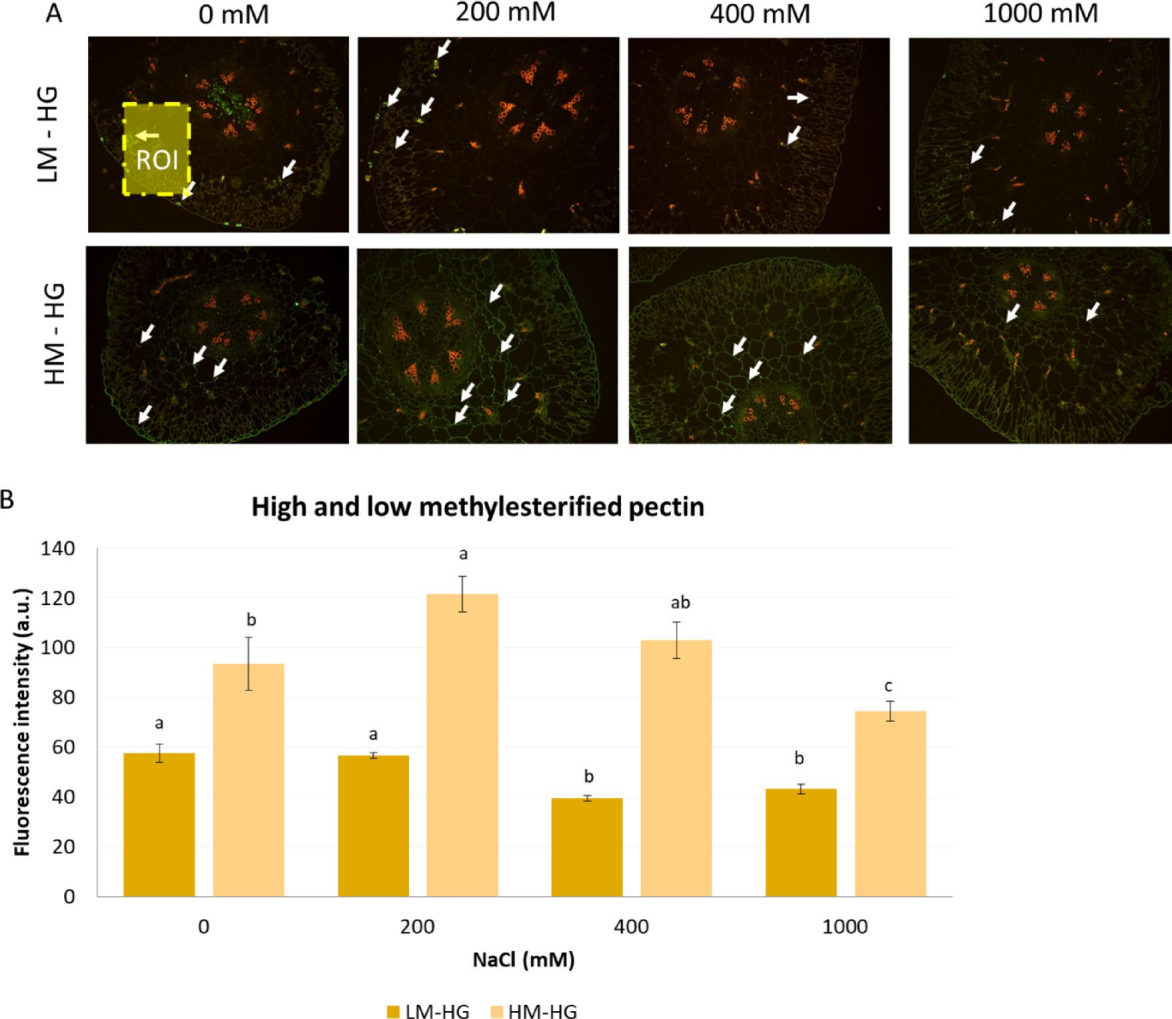
Distribution and quantification of high- and low-methylesterified homogalacturonan (HM- LM- HG) of *S. europaea* stem under different NaCl treatments from the cortical/parenchymatic ROI: region of interest, excluding vascular bundles. (**A**) Representative fluorescence microscopy images of cross-sections stained for LM-HG and HM-HG at 0, 200, 400, and 1000 mM NaCl. White arrows indicate regions of strong fluorescence intensity. (**B**) Quantification of fluorescence intensity for LM-HG and HM-HG across NaCl treatments. Data represent mean ± SD. *n* = 3 replicates per treatment. Different letters indicate statistically significant differences (*p* < 0.05).

Figure 3 presents the effect of NaCl-induced stress on the distribution and quantification of pectic homogalacturonan (HG) in plant cell walls. Figure 3A displays fluorescence microscopy images of cross-sections stained for low-methylesterified LM-HG, top row and HM-HG, bottom row at increasing NaCl concentrations (0, 200, 400, and 1000 mM). Figure 3B quantifies fluorescence intensity for LM-HG and HM-HG at different NaCl concentrations. At 0 mM NaCl, LM-HG fluorescence intensity was lower than that of HM-HG. At 200 mM NaCl, a significant increase in HM-HG fluorescence intensity was observed, while LM-HG remained unchanged. However, at 400 mM NaCl, LM-HG fluorescence intensity decreased significantly, whereas HM-HG remained high. At 1000 mM NaCl, LM-HG fluorescence was maintained low, while HM-HG decreased, suggesting a shift in pectin composition under severe salt stress. Different letters indicate statistically significant differences (*p* < 0.05) between treatments.

### Lignin localization and quantification

**Fig. 4:**
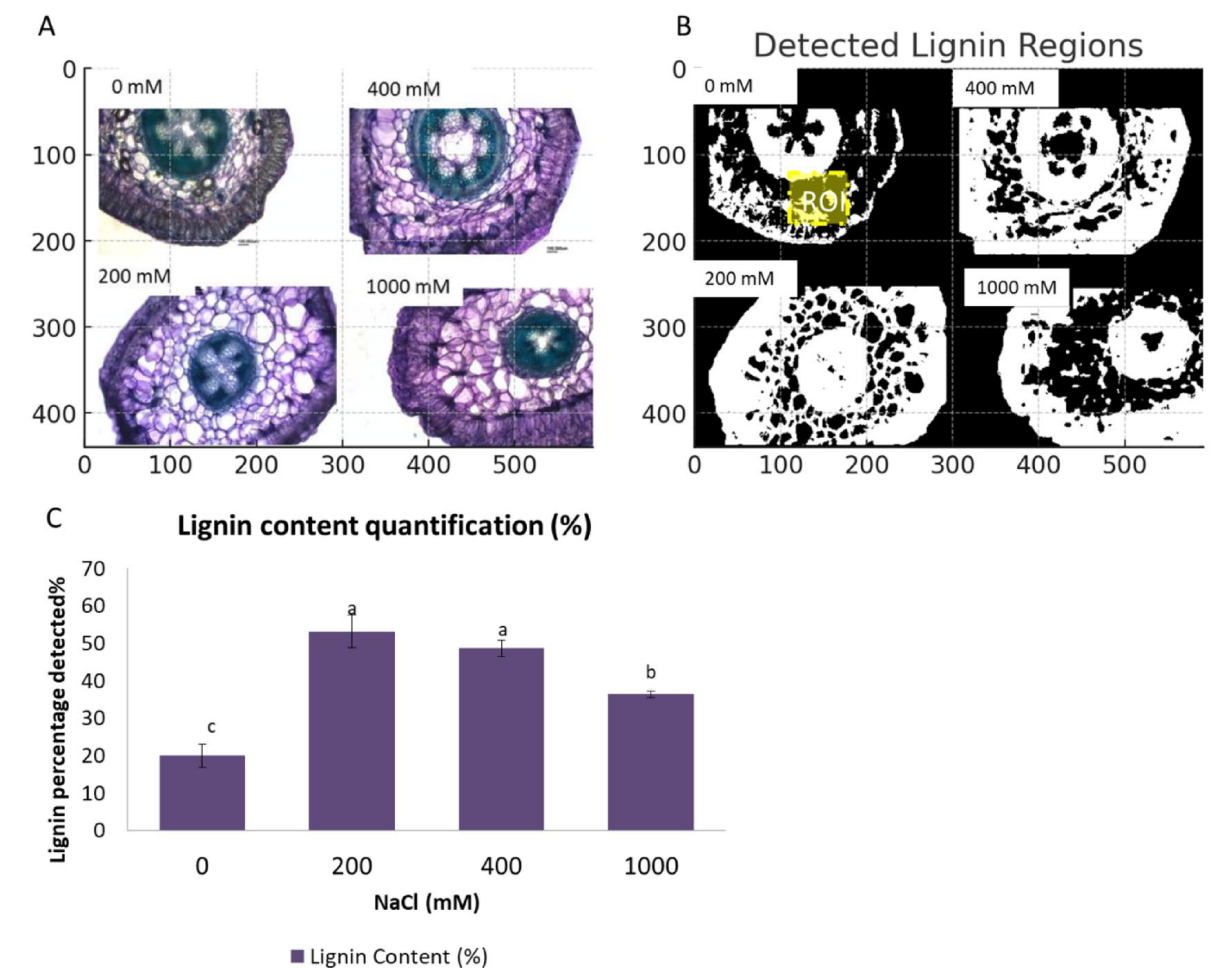
Microscopic histochemical detection and quantification of lignin deposition in *S. europaea* shoots under different salinity levels (0, 200, 400, and 1000 mM NaCl). (**A**) Cross-sectional images of *S. europaea* shoots stained with toluidine blue, where bluish-green regions indicate lignified tissues. (**B**) Binarized images highlight the detected lignin regions for quantitative analysis. ROI: cortical/parenchyma region of interest used for lignin quantification (shown as dashed yellow box) (**C**) Lignin content (%) quantified as the proportion of lignified area relative to total tissue area. Data represent mean ± SD. Statistical analysis with different letters indicates significant differences among treatments (*p* < 0.05). *n* = 3 replicates per treatment. Scale bar: 100 μm.

Microscopic analysis of *S. europaea* stem cross-sections stained with toluidine blue revealed distinct lignified regions, appearing bluish-green under different salinity levels (0, 200, 400, and 1000 mM NaCl) (Fig. 4A). Image processing and binarization identified and quantified lignin deposition, highlighting variations in lignified area across treatments (Fig. 4B). Quantitative analysis (Fig. 4C) showed a significant increase in the stained lignin content under salinity gradient. At 0 mM, lignin detection was sparse and confined to small areas (15%). However, at 200 and 400 mM NaCl compared to 0 mM, lignin staining was more widespread (both ~ 55%), particularly in vascular and peripheral tissues. At 1000 mM NaCl, lignin content identification (~ 40%) was reduced relatively to that at 200 and 400 mM, but remained evident in vascular regions and was higher than that at 0 mM.

### Lignin characterization by nitrobenzene oxidation yields

**Fig. 5:**
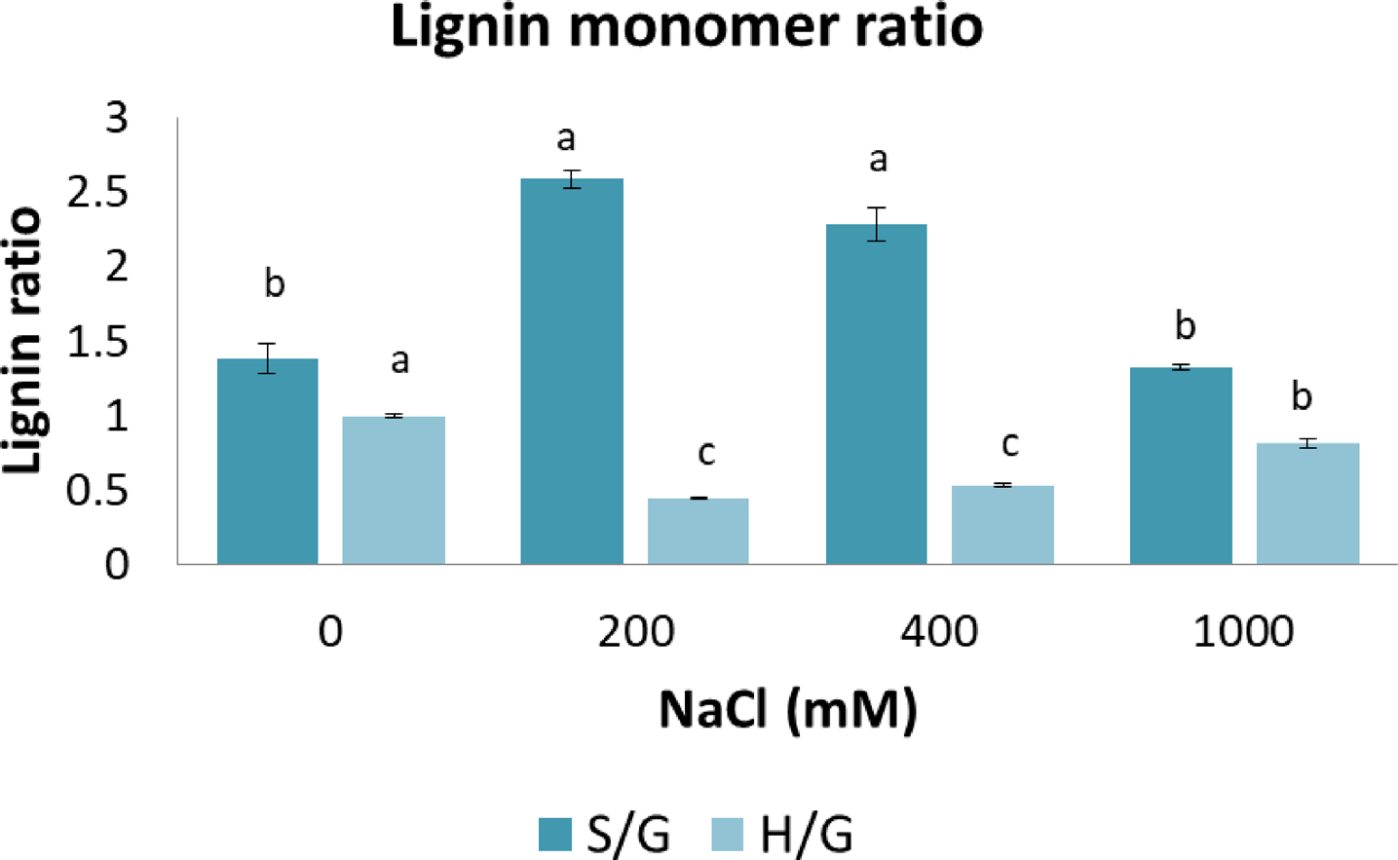
Lignin monomer ratios (S/G and H/G) in *S. europaea* shoots at different salinity levels (0, 200, 400, and 1000 mM NaCl). Data represent mean ± SD. Different letters within each group indicate significant differences at *p* < 0.05. *n* = 4 replicates per treatment.

Figure 5; Table 1 collectively illustrate the adaptive response of *Salicornia* to salinity stress, focusing on lignin monomer composition and yields of nitrobenzene oxidation products. The Table 1 provides quantitative data on lignin degradation products, while Fig. 5 shows the changes in lignin monomer ratios (S/G and H/G). The yields of S, G, and H monomer units varied with the salinity gradient (Table 1), reflecting the differential biosynthesis and degradation of lignin influenced by salinity.

**Table 1:** Monolignol yields of nitrobenzene oxidation products (% of oven dry extracted biomass). Data represent mean ± SD. Different letters indicate differences between treatments (*p* < 0.05).

Compound	Salinity treatments (mM)			
	0	200	400	1000
Syringic acid	0.18 ± 0.01 b	0.27 ± 0.04 a	0.20 ± 0.00 b	0.29 ± 0.02 a
Syringaldehyde	0.09 ± 0.01 d	0.77 ± 0.10 a	0.68 ± 0.02 b	0.22 ± 0.02 c
Vanillic acid	0.13 ± 0.02 b	0.10 ± 0.01 c	0.09 ± 0.02 c	0.20 ± 0.02 a
Vanillin	0.07 ± 0.00 c	0.31 ± 0.04 a	0.30 ± 0.01 a	0.18 ± 0.01 b
*p*-Hydroxybenzoic acid	0.08 ± 0.00 b	0.02 ± 0.00 c	0.02 ± 0.00 c	0.16 ± 0.03 a
*p*-Hydroxybenzaldehyde	0.10 ± 0.02 c	0.16 ± 0.00 b	0.19 ± 0.01 a	0.16 ± 0.01 b
Total yield	0.65 ± 0.01 d	1.62 ± 0.19 a	1.48 ± 0.06 b	1.21 ± 0.06 c

The increase in lignin deposition detected by light microscopy and image analysis at 200–400 mM NaCl in Fig. 4 was accompanied by the rise in S/G ratio (up to ~ 2.8), as seen in Fig. 5. Yields of H units increased at extreme salinity (1000 mM NaCl) (Table 1), aligning with a lowered S/G ratio as shown in Fig. 5, indicating a shift toward a more condensed lignin structure with a higher proportion of carbon–carbon linkages^11,52^.

### Shedding light on salt: polarized microscopy of intracellular accumulation pattern

**Fig. 6:**
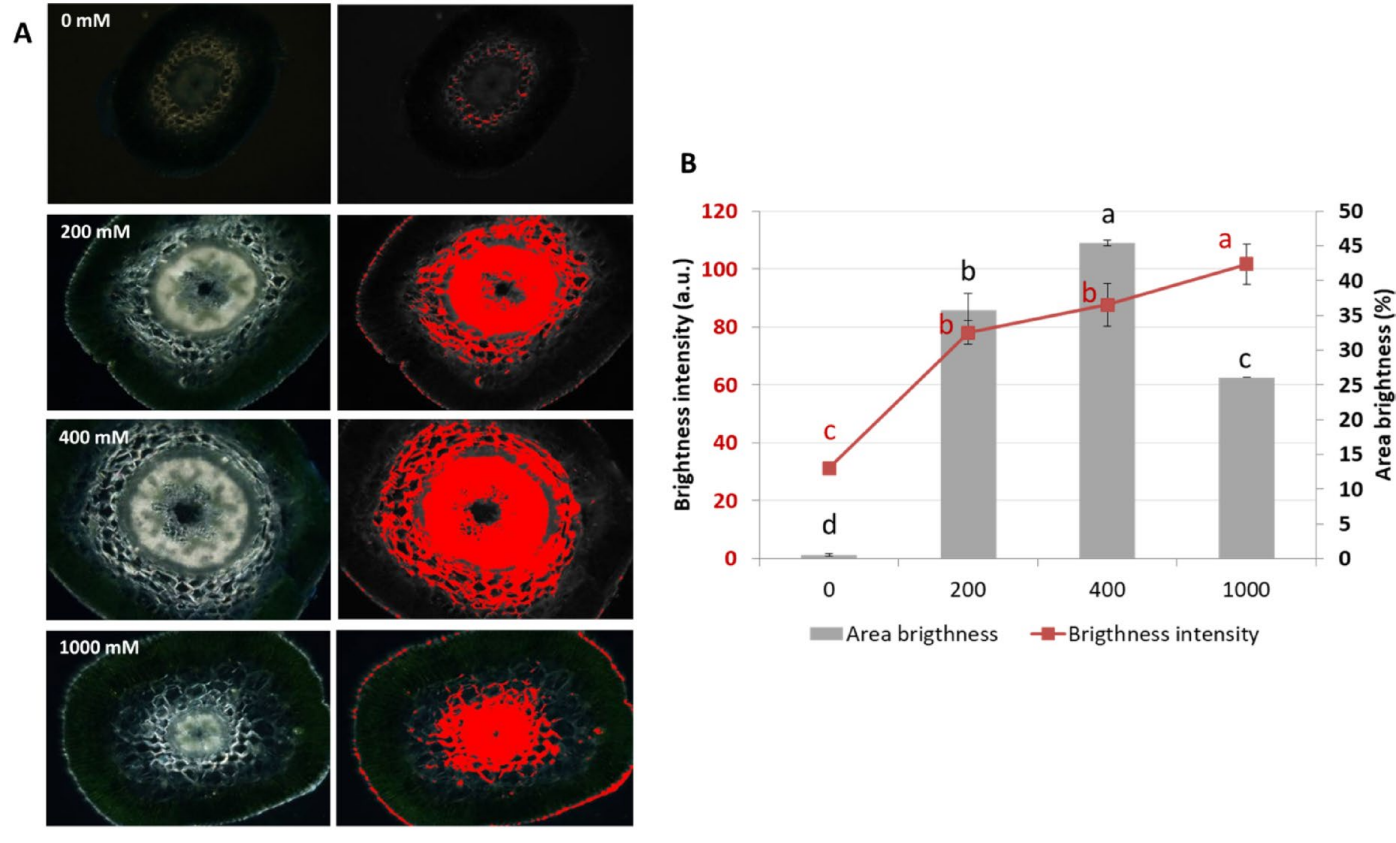
Effects of salinity on birefringence intensity and area coverage in *S. europaea* stem cross-sections. (**A**) Polarized light microscopy images of stem cross-sections exposed to different NaCl concentrations (0, 200, 400, and 1000 mM). The left column shows standard bright-field images, while the right column highlights birefringent regions (red overlay) detected under polarized light. (**B**) Quantification of birefringent area (gray bars, right y-axis) and birefringence intensity (red line, left y-axis) across treatments. Statistical differences among treatments are indicated by different letters. Data represent mean ± SD. Different letters indicate statistically significant differences between treatments (*p* < 0.05). *n* = 3 replicates per treatment.

Polarized light microscopy images of *Salicornia* stem cross-sections subjected to increasing NaCl concentrations show progressive changes in birefringence patterns across specific tissue regions (Fig. 6A–B). At 0 mM NaCl, minimal birefringence was detected, with low brightness intensity and area coverage. At 200 mM NaCl, birefringence increased markedly in the central parenchyma and vascular tissues, with the higher area brightness and intensity. At 400 mM NaCl, the birefringent area reaches its maximum, prominently spanning the central parenchyma, vascular bundles, and cortex regions, accompanied by increased brightness intensity. At 1000 mM NaCl, the birefringent area declined compared to 400 mM, while brightness intensity remained elevated, with signal concentrated in the central parenchyma and vascular tissues. Quantitative analysis (Fig. 6B) confirmed these patterns, showing significant increases in both area brightness (%) and brightness intensity (a.u.) from 0 mM to 400 mM, followed by reduced area brightness and sustained high intensity at 1000 mM.

**Fig. 7:**
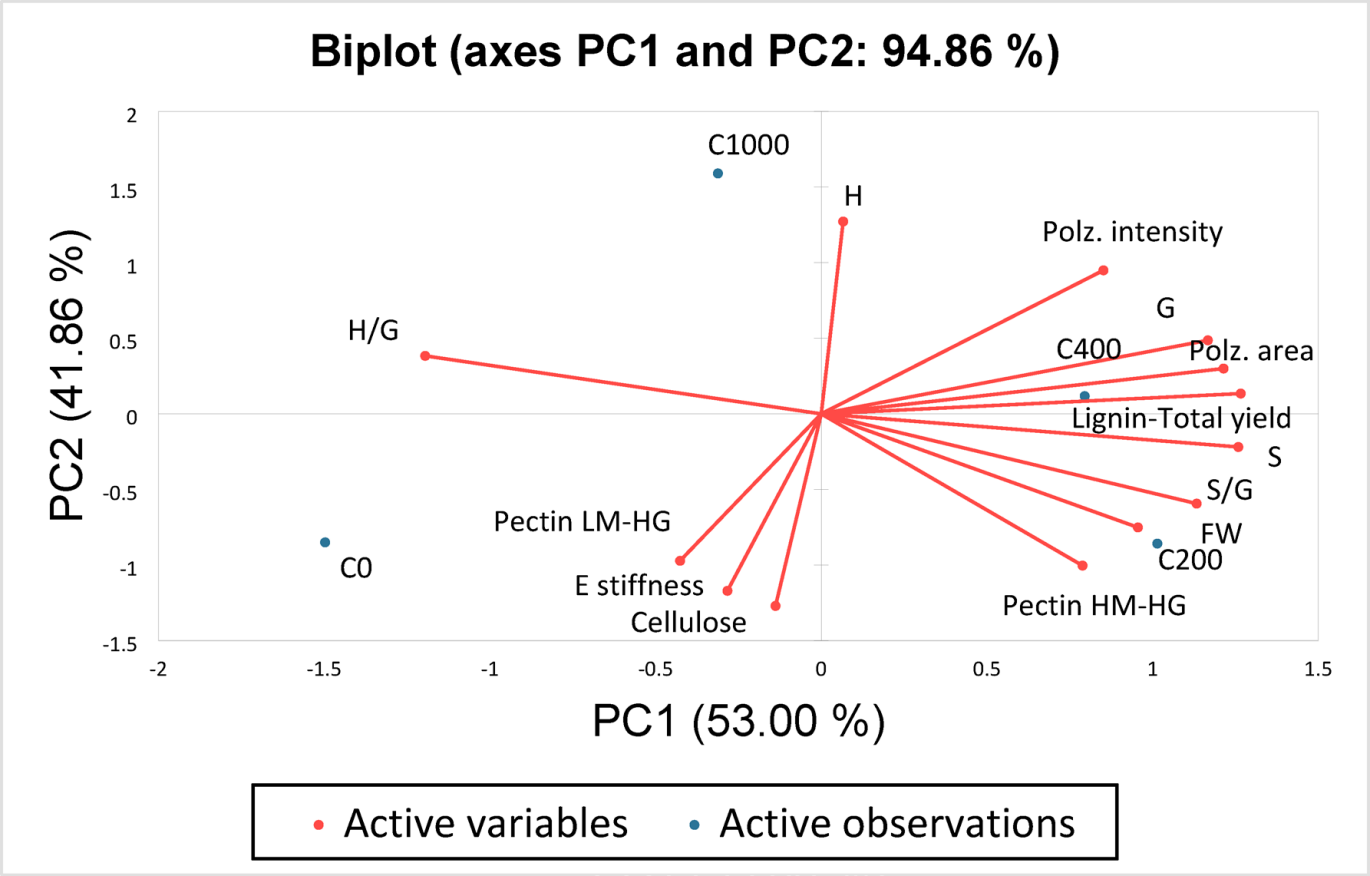
Principal Component Analysis (PCA) biplot displaying the first two principal components (PC1: 51.52% and PC2: 43.68%), which together explain 95.20% of the total variance. Active variables (red vectors) represent cell wall components and properties in *S. europaea* shoots, while active observations (blue points) correspond to different salinity-sample groups (C0, C1000, C400, C200). The direction and length of the vectors indicate the contribution and correlation of variables to the principal components. S: syringaldehyde + syringic acid, G: vanillin + vanillic acid, H: hydroxybenzaldehyde + *p*-hydroxybenzoic acid, *E*: Young’s modulus representing cell wall stiffness, FW: biomass fresh weight, Polz area: polarized brightness area and Polz. intensity: polarized btightness intensity.

In order to reduce the complexity of the dataset and to identify major axes of variation that explain coordinated cell wall structural traits and biochemical responses to increasing salinity, a PCA biplot was conducted (Fig. 7). The plot allowed the projection of active variables (in red) and treatment observations (in blue) onto the same ordination space. The two principal components (PC1 and PC2) accounted for 94.86% of the total variance, with PC1 explaining 53% and PC2 explaining 41.86%. This high cumulative variance indicated that the selected variables effectively described the observed variations among the samples.

## Discussion

### Cell wall nanomechanics: remodeling strategies

Salinity imposes osmotic and mechanical constraints, making cell wall nanomechanics a central adaptive trait^6^.

Our findings (Fig. 1), reinforce previous evidence that nanomechanical and physiological adaptations are central to salinity tolerance in *S. europaea*^2,24,53–55^. The progressive decrease in cell-wall stiffness with increasing NaCl in the Ciechocinek population in a nature reserve with high salinity (~ 100 dS m⁻¹), mirrors the pattern reported for the Inowrocław inland population associated with soda factory waste areas with lower salinity (~ 55 dS m⁻¹)^2,56^.

As shown in Cárdenas Pérez et al. ^2^ and Supplementary Table S1, reduced cellulose and lignin content coincided with a marked decline in *E*, indicating increased wall flexibility for osmotic adjustment under hypersaline conditions.

Notably, the Inowrocław population consistently exhibited higher biomass production and lower *E* values across all salinity treatments^2,24^ (Supplementary Table S1), suggesting a structural preadaptation that allows for sustained wall extensibility. In halophytes, such cell wall softening is thought to represent a key adaptive strategy, enhancing plasticity to accommodate water flux and maintain turgor under osmotic stress^57,58^.

By contrast, the Ciechocinek population showed delayed but pronounced wall softening, initially maintained higher cell wall stiffness at 0–400 mM NaCl, followed by a pronounced reduction in *E* at 1000 mM suggesting a population-specific strategy where rigidity is maintained until extreme stress triggers remodeling. This mechanical plasticity allows osmotic adjustment and water uptake under severe salt exposure.

These contrasting responses reveal population-specific strategies in *S. europaea*: Inowrocław relies on consistently softened walls, whereas Ciechocinek delays adjustment. Coordinated shifts in wall mechanics, cellular remodeling, and biomass (Supplementary Table S1) likely underlie their distinct salinity adaptation.

### Cell wall remodeling and reduced cellulose fluorescence in *S. europaea* water parenchyma tissue

The observed reduction in cellulose fluorescence at increasing NaCl concentrations (Fig. 1A–B) is consistent with salt-induced wall remodeling in halophytes^2,59^. Under saline conditions, plants modify their cell wall architecture decreasing cellulose deposition to enhance flexibility and maintain cellular integrity^1,2,53,55^. The significant decline in cellulose fluorescence at 400 mM NaCl across the water parenchyma tissue in R2 (intermediate parenchyma layer) and R3 (outer zone proximal to the cortex) compared to R1 (inner zone adjacent to the vascular bundle) suggests salinity-induced cell wall remodeling with variations in structural adaptations.

At 1000 mM NaCl, the further reduction in fluorescence intensity across the analysed regions indicates a severe disruption in cellulose biosynthesis, likely as an adaptive mechanism to enhance the cell wall plasticity and facilitate osmotic adjustments^60^. The pronounced decrease in R2 and R3 regions suggests that *S. europaea* prioritizes flexibility over rigidity, a trait commonly observed as more pronounced in species adapting to high ionic stress^2,9,55^.

The statistical differences support the hypothesis that salt stress progressively reduces cellulose rigidity, reflecting a fundamental trade-off between mechanical support and cellular expansion under saline conditions^61^. Cellulose correlated strongly with *E* (*r* = 0.95, *p* < 0.05; Supplementary Table S2), linking reduced deposition to lower wall stiffness and greater extensibility.

From a biofunctional standpoint, reduced fluorescence of cellulose under optimal to high salinity may reflect adjustments in wall architecture that favor increased matrix porosity, likely mediated by changes in pectin dynamics. This trait improves enzymatic accessibility during bioethanol production, reducing pretreatment costs. In food and nutraceuticals, decreased crystallinity of cellulose contributes to softer dietary fibers and improves prebiotic properties. Thus, *S. europaea*’s salt-responsive cellulose remodeling aligns with dual-use applications in functional food and biorefinery sectors. For instance, it has been reported that *Salicornia* sp. cellulose can be used as dietary fiber in foods, enhancing digestive health and functional formulations. In pharmaceuticals, it serves as an excipient and controlled-release agent in tablets and capsules, offering a sustainable plant-based source^62^.

Increased wall softening linked to specific cellulose profiles may enhance biomass processing for biofuels. The ability of *S. europaea* to adjust cellulose biosynthesis under extreme stress highlights its potential as a resilient crop source. Combined with previously reported high phenolic, vitamin, carotenoid, and polyphenol contents, which confer antioxidant, antimicrobial, and anti-inflammatory activities, these traits support its prospective use in pharmaceutical and nutraceutical applications, as documented in earlier studies^63^.

### Pectin methylesterification and salinity response

Pectin plays a key role in wall plasticity and stress adaptation. HM-HG enhances wall hydration and extensibility, whereas LM-HG can increase rigidity through Ca²⁺-mediated ‘egg-box’ cross-linking when de-esterification is block-wise, but may increase flexibility if random^64–66^. The results showed a significant increase in HM-HG fluorescence at optimal salinity (Fig. 2A-B) (200 and 400 mM NaCl), suggesting enhanced cell wall softening and flexibility to maintain turgor pressure and osmotic balance^61^. This observation is consistent with studies on halophytes demonstrating pectin modifications as a key adaptive strategy under salt stress^1^.

At 1000 mM NaCl, HM-HG fluorescence dropped to ~ 70 a.u. from its peak of ~ 120 a.u. at 200 mM, while LM-HG remained stable at ~ 45–50 a.u. (Fig. 3A–B). Letters in Fig. 3B confirm that HM-HG at 1000 mM was significantly lower than at 200 mM (*p* < 0.05). This quantitative decline in HM-HG pectin, together with the relative persistence of low-methylesterified forms, reflects a shift in pectin methylesterification dynamics rather than a net transition from HM- to LM-HG under severe ionic stress. While such changes can, under certain conditions, promote Ca²⁺-mediated cross-linking and strengthen cell wall gels, the concurrent reduction in both pectin fractions observed here is more consistent with a decrease in total pectin content or altered accessibility rather than extensive “egg-box” formation. These findings emphasize the importance of pectin methylesterification dynamics in modulating cell-wall integrity and mechanical properties, contributing to the plant’s response to high salinity^59,65^.

The ability to modulate pectin structure under varying salinity conditions may enhance *Salicornia* sp.‘s use in functional foods, offering improved textural properties and nutritional benefits.

Increased HM-HG at optimal salinity has translational value, enhancing gelling properties and bioactivity^67–69^. These attributes support the development of functional foods and plant-based therapeutics derived from *S. europaea*. At high salinity (1000 mM NaCl), the reduction in HM-HG and stabilization of LM-HG reflects a shift toward ionic cross-linking, which stabilizes wall structure but reduces biofunctionality.

In terms of bioconversion purposes, the reduction in LM-HG fluorescence at extreme salinity indicates that salt stress alters pectin composition, which can modulate cell wall accessibility. Although lignin is widely recognized as a major contributor to biomass recalcitrance, changes in pectin methylation under moderate salinity may facilitate hydrolysis and pretreatment efficiency^23,70^. HM-HG pectin at optimal salinity can reduce recalcitrance and enhance bioethanol yields, supporting the use of *Salicornia* sp. as a bioenergy crop^71,72^. These results underscore the complementary role of pectin remodeling alongside lignin dynamics in influencing saccharification efficiency.

### Lignin characterization in *S. europaea* under salinity stress

Lignin is essential for structural integrity, water transport, and stress resistance in plants^73,74^. Histochemical staining and quantification (Fig. 4A–C) showed clear salinity-driven changes. This semi-quantitative approach is sensitive to intensity shifts, however future Raman or FTIR analyses will refine these observations. At optimal salinity (200–400 mM NaCl), lignin staining intensity increased across several tissues, particularly in cortical/peripheral regions and around the vascular bundles (Fig. 4A). For quantitative analysis, cortical/parenchymatic tissues were considered (Fig. 4B), where a significant increase in lignified area was detected compared with the non-saline treatment^75,76^. Increased lignification under these conditions may also aid in maintaining vascular functionality by reinforcing xylem cell walls against salt-induced hydraulic stress.

At extreme salinity, lignin increased compared to the non-saline treatment but declined relative to optimal salinity, likely reflecting metabolic trade-offs that redirect energy toward osmolyte accumulation, ion compartmentalization, or antioxidant defense^1,77^. Consistent with this interpretation, Supplementary Table S2 shows a moderate positive correlation with total lignin (*r* = 0.535, *p* < 0.05), although this relationship was not statistically significant, and between HM-HG pectin and the S/G ratio (*r* = 0.907, *p* < 0.05), indicating that pectin remodeling is coordinated with lignin deposition under salinity stress.

Reduced lignification at high salinity relative to optimal salinity, while still remaining elevated compared to the non-saline treatment, may therefore signal not only a partial loss of structural support but also a regulated shift toward pectin-mediated ionic buffering and antioxidant functions, contributing to the observed decrease in cell-wall stiffness and biomass under extreme stress.

The observed increase in lignin content at 200–400 mM NaCl is consistent with previous studies showing that optimal salt stress induces lignin biosynthesis to reinforce cell walls, improving mechanical stability and limiting ion toxicity^60,78^. Lignin accumulation is an essential adaptive strategy, preventing excess Na⁺ infiltration and maintaining vascular function under osmotic stress^79^. The decline in total monolignol yield at severe salinity relative to optimal salinity levels (200–400 mM) suggests a threshold beyond which excessive ionic stress begins to inhibit lignin biosynthesis or oxidative release, even though the accumulation of lignin remains higher than in the non-saline control. This likely reflects that 0 mM NaCl is suboptimal for this halophyte, with low lignin driven by other factors. At high salinity, energy is redirected to osmoprotectant and antioxidant responses over wall reinforcement^75,80^. Under saline conditions, lignin distribution patterns gain particular histochemical significance for cell wall organization, reflecting stress-induced adjustments in structural integrity.

In this study, Toluidine Blue O was used as a general histological approach to distinguish tissues with differing degrees of lignification; however, this technique provides only preliminary anatomical information and does not allow lignin-specific detection or quantitative assessment. To achieve higher analytical resolution, future studies should complement and validate these observations using more specific methods such as the Wiesner (phloroglucinol-HCl) reaction for histochemical identification of lignin or lignin autofluorescence for quantitative imaging of its spatial distribution. Within the methodological scope of the present work, the observed patterns still reveal coordinated changes between lignin localization and pectin remodeling, suggesting a complementary contribution of both polymers to maintaining cell wall functionality and mechanical stability under ionic stress.

### Lignin structure and salt stress adaptation

Changes in S/G and H/G ratios in lignin (Fig. 5) indicate that salinity affects lignin polymerization patterns. Notably, lignins synthesized in response to environmental stresses have been shown to exhibit distinct structural features compared to those formed during normal development^81^. Optimal salinity (200–400 mM) promoted the accumulation of S-rich lignin, which is associated with more flexible and hydrophobic polymers that enhance water transport and mechanical performance under stress^16,75,82^. G units, typically linked to stronger cross-linking and rigidity^52^, increased less prominently than S units, indicating a shift toward a more elastic and less recalcitrant lignin. S units also contribute to ROS scavenging^83–85^. At 1000 mM NaCl, S/G and H/G ratios returned toward non-saline levels, indicating reduced lignification under extreme stress. The relative increase in H units may reflect a minimal structural maintenance strategy. The effect of S/G and H/G shifts may vary across species^20^.

Similar patterns occur in halophytes and glycophytes under high salinity, where excess NaCl inhibits the phenylpropanoid pathway and redirects energy toward osmotic regulation and stress responses^1,86^.

Lignin content and monomer composition in *S. europaea* respond dynamically to salinity: optimal NaCl stimulates lignin deposition and S/G shifts supporting vascular reinforcement, while 1000 mM reduces lignin and reverts profiles toward non-saline levels. This likely reflects resource diversion to osmolyte and antioxidant defenses. These correlations highlight lignin’s adaptive role but warrant further molecular validation.

Lignin modulation in *S. europaea* under salinity offers applications in food, pharma, and biofuels. In food, increased lignin at optimal salinity enhances fiber and bioactive content. S-rich lignins provide antioxidant, antimicrobial, and anti-inflammatory phenolics with potential for drug delivery and cell protection^87^. These can be extracted for use in therapeutic formulations. In contrast, the increased presence of *p*-hydroxyphenyl (H) units under extreme salinity (1000 mM NaCl) coincided with the sharp decline in *E*, and correlation analysis confirmed a strong negative relationship between H content and *E* (*r* = − 0.89, *p* < 0.05). This pattern reflects a coordinated remodeling of the cell wall matrix rather than a simple enrichment of specific lignin units. Under severe salt stress, increased lignin yield combined with reduced cellulose rigidity and altered pectin composition leads to a less integrated and more compliant wall structure. The relative contribution of H units may represent a potential cost-saving strategy, as their biosynthesis requires fewer enzymatic steps than G or S units^11,16,73,88^, allowing the plant to maintain minimal structural functionality while prioritizing stress tolerance. While potentially less suitable for biofuel conversion, this adaptation allows survival under high stress by conserving energy for osmoprotection and ion homeostasis. For biofuels, the shift toward S-rich lignin at optimal salinity facilitates more efficient lignocellulosic bioethanol production, while reduced lignin content at extreme salinity lowers biomass recalcitrance, improving biofuel processing efficiency. These findings highlight *S. europaea* as a sustainable multipurpose crop with applications in health and food industries, as well as a promising feedstock to biofuel conversion^89^.

Supplementary Table S2 shows no significant correlation between total lignin and *E* (*r* = − 0.349), indicating lignin amount alone does not determine stiffness. *E* correlated strongly negatively with H units (*r* = − 0.889, *p* < 0.05) and weakly with H/G, while S and G units correlated positively with total lignin (*r* = 0.961 and 0.958).

These patterns suggest that lignin monomer shifts, particularly H enrichment at 1000 mM NaCl, are more closely linked to reduced wall stiffness than total lignin, indicating monomeric remodeling drives mechanical adjustment under extreme salinity.

### Polarized microscopy for mapping salinity effects in plant tissues

Polarized light microscopy (PLM) revealed progressive changes in salt-related birefringence in *S. europaea* across salinity levels. At 0 mM NaCl, birefringence was minimal, whereas at 200–400 mM both brightness intensity and bright area increased, indicating active salt sequestration in parenchyma tissues^48^. At 1000 mM NaCl, intensity remained high but localized, with a sharp decline in bright area (Fig. 6A–B), suggesting denser deposits in peripheral regions near the pith cylinder and epidermis^90^. These changes imply cell-wall remodeling through lignification, polysaccharide rearrangement, or salt binding^91^, consistent with known halophyte ion sequestration strategies^49,59,92^. Excessive NaCl can disrupt biopolymer networks and reduce wall extensibility^93^; the maintained intensity despite reduced area may reflect densification of birefringent components.

Birefringence may also reflect transitions in the physical state of accumulated salts (e.g., crystallization–dissolution cycles) and ion redistribution between intra- and extracellular compartments, governed by local salinity and tissue hydration. The PLM approach is consistent with prior work in halophytes (*Salicornia bigelovii*)^94^ and can be complemented with Raman spectroscopy or related methods to resolve salt composition and localization in future studies.

### Coordination of nanomechanics and polymer remodeling

When the nanomechanical and biochemical data are viewed together, they reveal a coordinated adjustment between cell-wall stiffness *E* and polymer composition a pattern consistent with broader concepts of stress-induced cell wall remodeling described in recent reviews^95^. Across all treatments, changes in *E* closely followed changes in cellulose (*r* = 0.95) and showed a moderate association with HM-HG pectin (*r* = 0.56), while being negatively related to H-type lignin (*r* = − 0.89), polarized-light area (*r* = − 0.35), and polarized-light intensity (*r* = − 0.85) (Supplementary Table S2). Similar relationships between mechanical stiffness and polysaccharide composition under salt stress have been reported in maize and other glycophytes^75^.

The strong link with cellulose indicates that changes in cellulose organization rather than a reduction in its amount were a key factor driving the decline in wall stiffness under optimal salinity. The lower fluorescence intensity likely reflects increased wall flexibility and structural rearrangements associated with tissue expansion and a higher number of parenchyma cells. High HM-HG pectin levels combined with lower H-lignin proportions at this range reflect looser, more hydrated cell walls with greater ionic buffering capacity. Such dynamic adjustments of pectin and lignin to maintain wall hydration and flexibility are characteristic adaptive features of plants exposed to optimal salt stress^76^.

At 1000 mM, *E* remained low, correlating negatively with H-lignin (*r* = − 0.89), G units (*r* = − 0.60) and polarized-light intensity (*r* = − 0.85), but positively with cellulose (*r* = 0.95) and HM-HG pectin (*r* = 0.56). Cellulose strongly anti-correlated with H (*r* = − 0.99). H enrichment is linked to lower biosynthetic cost and weaker mechanics under stress^11,16,76^. Although both G and S monomers increased proportionally with total lignin content at high salinity, this did not translate into higher wall stiffness, suggesting that the mechanical response is more closely linked to local cell wall architecture and polymer interactions than to overall lignin quantity.

Polarized-light microscopy supports these patterns: at optimal salinity, decreased birefringence area and increased peripheral brightness paralleled lower *E*, reduced cellulose, and higher HM-HG pectin, indicating ion compartmentalization and hydration act with polymer remodeling to maintain turgor and flexibility^48^. At 1000 mM, birefringence became more localized but very intense, consistent with low *E*, low HM-HG pectin, and stronger structural reinforcement.

The PCA (Fig. 7) shows E clustering with LM-HG and cellulose on the negative PC1 side, while HM-HG pectin, S/G ratio, biomass, lignin traits, and polarized-light parameters load positively, highlighting their link to wall elasticity under optimal salinity.

The PCA biplot (94.86% variance) shows clear treatment separation. Optimal salinity (200–400 mM) clusters with higher biomass, cellulose, lignin, S/G ratio, HM-HG pectin, and polarized-light area, indicating wall loosening, osmotic buffering, and water retention.

Non-saline treatment aligns with higher stiffness, cellulose, and H/G ratio, reflecting more rigid, G-rich lignin, whereas 1000 mM groups with H units and polarized-light intensity, indicating reduced biomass and less integrated wall structure. The separation of H/G from S/G and total lignin highlights a trade-off between metabolic cost and structural flexibility.

These results show that *S. europaea* displays finely tuned, salinity-dependent cell wall remodeling, integrating stiffness, polymer composition, and biomass performance as part of its adaptive strategy.

### Integrated polymer applications across salinity gradient

*S. europaea* adapts to optimal salinity (200–400 mM) through increased methyl-esterified pectin, enhancing wall plasticity, hydration, and osmotic balance. Salinity modulates polymers in a complementary manner: cellulose becomes structurally less rigid, contributing to greater flexibility; pectin remodels to improve hydration and porosity; and lignin shifts to maintain structural balance and energy allocation. These coordinated adjustments highlight promising biotechnological applications, particularly for improving biomass accessibility and conversion efficiency, and warrant further experimental validation (Table 2).

**Table 2:** Potential applications of *S. europaea* cell wall polysaccharides at different salinity levels.

Salinity level (NaCl)	Cell wall biopolymer	Salinity-induced modifications	Potential applications	References
Low salinity (< 200 mM)	Pectin	Lower methylesterification; less plasticity	Limited texturizing or gelling use in food; lower nutraceutical value, lower bioactivity	^68^
	Cellulose	Moderate to deposition and increased stiffness; rigid wall architecture	Fiber source for food; limited flexibility for biofuel saccharification	^28^
	Lignin	Lower total lignin content and reduced abundance of both G and S units, reflecting limited lignification under suboptimal conditions	Lower structural reinforcement; reduced contribution to recalcitrance	^2,19^
Optimal salinity (200–400 mM)	Pectin	Increased methylesterification; enhanced plasticity, water retention	Food: improved gelling and thickening; Pharmaceuticals: immune modulation, gut health benefits	^31,63,68^
	Cellulose	Stable cellulose deposition with increased flexibility	Functional prebiotic food fiber with better digestibility; easier saccharification for bioethanol	^28,96^
	Lignin	Increased lignification and higher S/G ratio, reflecting enhanced deposition of S and G units at optimal salinity	Enhanced biofuel conversion, enzymatic saccharification; production with reduced energy input	^19,20^
High salinity (> 400–1000 mM)	Pectin	Decline in methylesterification; potential cross-linking	Reduced gelling quality; structural stabilization, less suitable for premium food uses	^63^
	Cellulose	Potential reduction in synthesis; structural weakening	Lower biomass yield; limited industrial application	^96^
	Lignin	Intermediate lignin content with higher H contribution relative to optimal salinity 200–400 mM, stable S/G and H/G ratios compared to non-saline, and altered matrix organization	Variable impact on biomass digestibility; potentially more recalcitrant	^19,20^

## Conclusions

This study uncovers a coordinated, salinity-responsive remodeling of pectin and lignin and a quantitative modulation of cellulose rigidity in *S. europaea*, revealing its structural plasticity and metabolic adaptability. At optimal salinity (200–400 mM NaCl), increased pectin methylesterification and elevated syringyl/guaiacyl (S/G) lignin ratios enhance wall flexibility, osmotic buffering, and apoplastic transport. Simultaneously, moderate reductions in cellulose deposition reduce wall rigidity, improving biomass accessibility.

Under extreme salinity, structural integrity is preserved through modified lignin composition, with a higher proportion of *p*-hydroxyphenyl (H) units compared to both non-saline and optimal salinity treatments, reflecting a shift toward a metabolically simpler lignin profile, and localized salt compartmentalization, revealing energy-saving adaptations. These polymer-specific responses reveal how *S. europaea* maintains structural and osmotic balance in hypersaline environments.

Importantly, these compositional shifts confer application-specific benefits: HM-pectin for food and pharmaceutical formulations, S-rich lignin for efficient enzymatic hydrolysis in biofuel production, and softened cellulose networks for increased digestibility. These findings position *S. europaea* as a promising multipurpose crop for saline agriculture, integrating stress resilience with high-value biopolymer yield.

## Supplementary Information

Below is the link to the electronic supplementary material.

Supplementary Material 1

## Data Availability

All data generated or analysed during this study are included in this published article.

## References

[1] Zhao, C., Zhang, H., Song, C., Zhu, J. K. & Shabala, S. Mechanisms of plant responses and adaptation to soil salinity. *Innov* **1**, 100017 (2020). 10.1016/j.xinn.2020.100017 PMC8454569 34557705

[2] Cárdenas Pérez, S. et al. Salinity-driven changes in salicornia cell wall nanomechanics and lignin composition. *Environ. Exp. Bot.* **218**, 105606 (2024).

[3] Burton, R. A., Gidley, M. J. & Fincher, G. B. Heterogeneity in the chemistry, structure and function of plant cell walls. *Nat. Chem. Biol.* **6**, 724–732 (2010). 20852610 10.1038/nchembio.439

[4] Cosgrove, D. J. Plant expansins: diversity and interactions with plant cell walls. *Curr. Opin. Plant. Biol.* **25**, 162–172 (2015). 26057089 10.1016/j.pbi.2015.05.014 PMC4532548

[5] Barbez, E., Dünser, K., Gaidora, A., Lendl, T. & Busch, W. Auxin steers root cell expansion via apoplastic pH regulation in Arabidopsis Thaliana. *Proc. Natl. Acad. Sci. U S A*. **114**, E4884–E4893 (2017). 28559333 10.1073/pnas.1613499114 PMC5474774

[6] Mendis, C. L., Padmathilake, R. E. & Attanayake, R. N. Learning from salicornia: Physiological, Biochemical, and molecular mechanisms of salinity tolerance. *MDPI Mol. Sci. MDPI* 3–8 (2025). 10.3390/ijms26135936 PMC12250511 40649711

[7] Tenhaken, R. Cell wall remodeling under abiotic stress. *Front. Plant. Sci.* **5**, 1–9 (2015). 10.3389/fpls.2014.00771 PMC4285730 25709610

[8] Cosgrove, D. J. Growth of the plant cell wall. *Nat. Rev. Mol. Cell Biol.* 6, 850–861 (2005). (2005). 10.1038/nrm1746 16261190

[9] Liu, J. et al. Cell wall components and extensibility regulate root growth in Suaeda Salsa and spinacia Oleracea under salinity. *MDPI Plants*. **11**, 1–13 (2022). 10.3390/plants11070900 PMC9002714 35406880

[10] Mohnen, D. Pectin structure and biosynthesis. *Curr. Opin. Plant. Biol.* **11**, 266–277 (2008). 18486536 10.1016/j.pbi.2008.03.006

[11] Vanholme, R., Demedts, B., Morreel, K., Ralph, J. & Boerjan, W. Lignin biosynthesis and structure. *Plant. Physiol.* **153**, 895–905 (2010). 20472751 10.1104/pp.110.155119 PMC2899938

[12] Liu, Q., Luo, L., Zheng, L. & Lignins Biosynthesis and biological functions in plants. *Int J. Mol. Sci* **19**, (2018). 10.3390/ijms19020335 PMC5855557 29364145

[13] Cybulska, I. et al. Chemical characterization and hydrothermal pretreatment of salicornia bigelovii straw for enhanced enzymatic hydrolysis and bioethanol potential. *Bioresour Technol.* **153**, 165–172 (2014). 24362358 10.1016/j.biortech.2013.11.071

[14] Santos, R. B., Capanema, E. A., Balakshin, M. Y., Chang, H. M. & Jameel, H. Lignin structural variation in hardwood species. *J. Agric. Food Chem.* **60**, 4923–4930 (2012). 22533315 10.1021/jf301276a

[15] Lourenço, A. et al. Biomass production of four Cynara cardunculus clones and lignin composition analysis. *Biomass Bioenerg.* **76**, 86–95 (2015).

[16] Han, X. et al. Lignin biosynthesis and accumulation in response to abiotic stresses in Woody plants. *For Res* **2**, (2022). 10.48130/FR-2022-0009 PMC11524291 39525415

[17] Bose, S. K., Francis, R. C., Govender, M., Bush, T. & Spark, A. Bioresource technology lignin content versus syringyl to guaiacyl ratio amongst poplars. *Bioresour Technol.* **100**, 1628–1633 (2009). 18954979 10.1016/j.biortech.2008.08.046

[18] Dumitrache, A. et al. Consolidated bioprocessing of Populus using clostridium (Ruminiclostridium) thermocellum: A case study on the impact of lignin composition and structure. *Biotechnol. Biofuels*. **9**, 1–14 (2016). 26855670 10.1186/s13068-016-0445-x PMC4743434

[19] Ďurkovič, J., Kačík, F., Husárová, H., Mamoňová, M. & Čaňová, I. Cell wall compositional and vascular traits of hybrid Poplar wood in micropropagated plants and plants propagated from root cuttings. *New. For.* **51**, 119–135 (2020).

[20] Yoo, C. G. et al. Significance of lignin S/G ratio in biomass recalcitrance of Populus trichocarpa variants for bioethanol production. *ACS Sustain. Chem. Eng.* **6**, 2162–2168 (2018).

[21] Van Eeckhout, A. et al. Polarimetric imaging microscopy for advanced inspection of vegetal tissues. *Sci. Rep.* **11**, 1–12 (2021). 33594126 10.1038/s41598-021-83421-8 PMC7887219

[22] Orzoł, A. et al. The local environment influences salt tolerance differently in four salicornia Europaea L. inland populations. *Sci. Rep.* **15**, 1–13 (2025). 40240466 10.1038/s41598-025-97394-5 PMC12003738

[23] Cárdenas Pérez, S., Niedojadło, K., Mierek-Adamska, A., Dąbrowska, G. B. & Piernik, A. Maternal salinity influences anatomical parameters, pectin content, biochemical and genetic modifications of two salicornia Europaea populations under salt stress. *Sci. Rep.* **12**, 1–16 (2022). 35194050 10.1038/s41598-022-06385-3 PMC8863803

[24] Cárdenas Pérez, S., Grigore, M. N. & Piernik, A. Prediction of salicornia Europaea L. biomass using a computer vision system to distinguish different salt-tolerant populations. *BMC Plant. Biol* **24**, (2024). 10.1186/s12870-024-05743-9 PMC11568609 39548379

[25] Gallegos-Cerda, S. D. et al. Decoding salinity tolerance in salicornia Europaea L.: Image-Based oxidative phenotyping and histochemical mapping of pectin and lignin. 1–23 (2025). 10.3390/plants14193055 PMC12526113 41095196

[26] El-Keblawy, A., Gairola, S. & Bhatt, A. Maternal salinity environment affects salt tolerance during germination in anabasis setifera: A facultative desert halophyte. *J. Arid Land.* **8**, 254–263 (2016).

[27] Lee, J. H. et al. The beneficial effect of salicornia herbacea extract and Isorhamnetin-3-O-glucoside on obesity. *Processes* **11**, 1–13 (2023).

[28] Cárdenas-Pérez, S., Piernik, A., Chanona-Pérez, J. J., Grigore, M. N. & Perea-Flores, M. J. An overview of the emerging trends of the salicornia L. genus as a sustainable crop. *Environ Exp. Bot* **191**, (2021).

[29] Lu, C., Napier, J. A., Clemente, T. E. & Cahoon, E. B. New frontiers in oilseed biotechnology: meeting the global demand for vegetable oils for food, feed, biofuel, and industrial applications. *Curr. Opin. Biotechnol.* **22**, 252–259 (2011). 21144729 10.1016/j.copbio.2010.11.006

[30] Katel, S., Yadav, S. P. S., Oli, S., Adhikari, R. & Shreeya, S. Exploring the potential of salicornia: A halophyte’s impact on Agriculture, the Environment, and the economy. *Peruv. J. Agron.* **7**, 220–238 (2023).

[31] Patel, S. & Salicornia Evaluating the halophytic extremophile as a food and a pharmaceutical candidate. *3 Biotech.* **6**, 1–10 (2016). 10.1007/s13205-016-0418-6 PMC4835422 28330174

[32] Wilkoń-Michalska, J. Zmiany Sukcesyjne w rezerwacie halofitów Ciechocinek w Latach 1954-1965. *Ochr Przyr Zakład Ochr Przyr PAN*. **1970** (R. 35), 1970 (1921-2005).

[33] Lubińska-Mielińska, S. et al. Inland salt marsh habitat restoration can be based on artificial flooding. *Glob Ecol. Conserv* **34**, (2022).

[34] Cárdenas-Pérez, S. et al. Image and fractal analysis as a tool for evaluating salinity growth response between two salicornia Europaea populations. *BMC Plant. Biol* **20**, (2020). 10.1186/s12870-020-02633-8 PMC7549212 33045997

[35] Bruker NanoWizard ^®^ AFM Handbook. *JPK Instruments Tech. Note, Berlin, Ger.* (2012).

[36] Hutter, J. L. & Bechhoefer, J. Calibration of atomic-force microscope tips. *Rev. Sci. Instrum.* **64**, 1868–1873 (1993).

[37] Routier-Kierzkowska, A. L. et al. Cellular force microscopy for in vivo measurements of plant tissue mechanics. *Plant. Physiol.* **158**, 1514–1522 (2012). 22353572 10.1104/pp.111.191460 PMC3343728

[38] Cárdenas-Pérez, S. et al. Nanoindentation study on Apple tissue and isolated cells by atomic force microscopy, image and fractal analysis. *Innov. Food Sci. Emerg. Technol.* **34**, 234–242 (2016).

[39] Kitin, P., Nakaba, S., Hunt, C. G., Lim, S. & Funada, R. Direct fluorescence imaging of lignocellulosic and suberized cell walls in roots and stems. *AoB Plants*. **12**, 1–19 (2020). 10.1093/aobpla/plaa032 PMC7415075 32793329

[40] Anderson, C. T., Carroll, A., Akhmetova, L. & Somerville, C. Real-time imaging of cellulose reorientation during cell wall expansion in Arabidopsis roots. *Plant. Physiol.* **152**, 787–796 (2010). 19965966 10.1104/pp.109.150128 PMC2815888

[41] Herburger, K. & Holzinger, A. Aniline blue and calcofluor white staining of Callose and cellulose in the streptophyte green algae zygnema and klebsormidium. *Bio-Protocol* **6**, 6–10 (2016). 10.21769/BioProtoc.1969 PMC5076763 27785458

[42] Goh, T. Y., Basah, S. N., Yazid, H., Aziz Safar, M. J. & Ahmad Saad, F. Performance analysis of image thresholding: Otsu technique. *Meas. J. Int. Meas. Confed*. **114**, 298–307 (2018).

[43] Niedojadło, K., Hyjek, M. & Bednarska-Kozakiewicz, E. Spatial and Temporal localization of homogalacturonans in hyacinthus orientalis L. ovule cells before and after fertilization. *Plant. Cell. Rep.* **34**, 97–109 (2015). 25292437 10.1007/s00299-014-1690-8 PMC4282716

[44] Pradhan Mitra, P. & Loqué, D. Histochemical staining of Arabidopsis Thaliana secondary cell wall elements. *J. Vis. Exp.* 1–11. 10.3791/51381 (2014). 10.3791/51381 PMC4186213 24894795

[45] Sluiter, A., Ruiz, R., Scarlata, C., Sluiter, J. & Templeton, D. Determination of Extractives in Biomass: Laboratory Analytical Procedure (LAP); Issue Date 7/17/2005. (2008).

[46] Kačíková, D., Kubovský, I., Ulbriková, N. & Kačík, F. The impact of thermal treatment on structural changes of Teak and Iroko wood lignins. *Appl Sci* **10**, (2020).

[47] Vermaas, J. V. et al. Passive membrane transport of lignin-related compounds. *Proc. Natl. Acad. Sci. U. S. A.* 116, 23117–23123 (2019). 10.1073/pnas.1904643116 PMC6859372 31659054

[48] Lv, S. et al. Multiple compartmentalization of sodium conferred salt tolerance in salicornia Europaea. *Plant. Physiol. Biochem.* **51**, 47–52 (2012). 22153239 10.1016/j.plaphy.2011.10.015

[49] Flowers, T. J. & Colmer, T. D. Salinity tolerance in halophytes. *New. Phytol*. **179**, 945–963 (2008). 18565144 10.1111/j.1469-8137.2008.02531.x

[50] SigmaPlot Systat Software Inc - SigmaPlot. (2013). http://www.sigmaplot.co.uk/

[51] XLSTAT. XLSTAT 2023 1.4 Basic | Software estadístico Excel. (2023). https://www.xlstat.com/en

[52] Boerjan, W., Ralph, J. & Baucher, M. Lignin biosynthesis. *Annu. Rev. Plant. Biol.* **54**, 519–546 (2003). 14503002 10.1146/annurev.arplant.54.031902.134938

[53] Beauzamy, L., Derr, J. & Boudaoud, A. Quantifying hydrostatic pressure in plant cells by using indentation with an atomic force microscope. *Biophys. J.* **108**, 2448–2456 (2015). 25992723 10.1016/j.bpj.2015.03.035 PMC4457008

[54] Feng, W. et al. The FERONIA receptor kinase maintains Cell-Wall integrity during salt stress through Ca2 + Signaling. *Curr. Biol.* **28**, 666–675e5 (2018). 29456142 10.1016/j.cub.2018.01.023 PMC5894116

[55] Colin, L. et al. The cell biology of primary cell walls during salt stress. *Plant. Cell.* **35**, 201–217 (2023). 36149287 10.1093/plcell/koac292 PMC9806596

[56] Piernik, A., Hulisz, P. & Rokicka, A. Micropattern of halophytic vegetation on technogenic soils affected by the soda industry. *Soil. Sci. Plant. Nutr.* **61**, 98–112 (2015).

[57] Cosgrove, D. J. Diffuse growth of plant cell walls. *Plant. Physiol.* **176**, 16–27 (2018). 29138349 10.1104/pp.17.01541 PMC5761826

[58] Cosgrove, D. J. & Jarvis, M. C. Comparative structure and biomechanics of plant primary and secondary cell walls. *Front. Plant. Sci.* **3**, 1–7 (2012). 22936943 10.3389/fpls.2012.00204 PMC3424969

[59] Vicré, M., Farrant, J. M. & Driouich, A. Insights into the cellular mechanisms of desiccation tolerance among angiosperm resurrection plant species. *Plant. Cell. Environ.* **27**, 1329–1340 (2004).

[60] Munns, R. & Tester, M. Mechanisms of salinity tolerance. *Annu. Rev. Plant. Biol.* **59**, 651–681 (2008). 18444910 10.1146/annurev.arplant.59.032607.092911

[61] Le Gall, H. et al. Cell wall metabolism in response to abiotic stress. *MDPI Plants*. **4**, 112–166 (2015). 10.3390/plants4010112 PMC4844334 27135320

[62] Lopes, M., Sanches-Silva, A., Castilho, M., Cavaleiro, C. & Ramos, F. Halophytes as source of bioactive phenolic compounds and their potential applications. *Crit. Rev. Food Sci. Nutr.* **63**, 1078–1101 (2023). 34338575 10.1080/10408398.2021.1959295

[63] Castagna, A. et al. Nutritional Composition and Bioactivity of Salicornia europaea L. Plants Grown in Monoculture or Intercropped with Tomato Plants in Salt-Affected Soils. *Horticulturae* 8, (2022).

[64] Wolf, S., Mouille, G. & Pelloux, J. Homogalacturonan methyl-esterification and plant development. *Mol. Plant.* **2**, 851–860 (2009). 19825662 10.1093/mp/ssp066

[65] Caffall, K. H. & Mohnen, D. The structure, function, and biosynthesis of plant cell wall pectic polysaccharides. *Carbohydr. Res.* **344**, 1879–1900 (2009). 19616198 10.1016/j.carres.2009.05.021

[66] Willats, W. G. T., Knox, J. P., Mikkelsen, J. D. & Pectin New insights into an old polymer are starting to gel. *Trends Food Sci. Technol.* **17**, 97–104 (2006).

[67] Yapo, B. M. Pectic substances: from simple pectic polysaccharides to complex pectins - A new hypothetical model. *Carbohydr. Polym.* **86**, 373–385 (2011).

[68] Lara-Espinoza, C., Carvajal-Millán, E., Balandrán-Quintana, R. & López-Franco, Y. & Rascón-Chu, A. Pectin and pectin-based composite materials: beyond food texture. *MDPI Mol* **23**, (2018). 10.3390/molecules23040942 PMC6017442 29670040

[69] Maxwell, E. G., Belshaw, N. J., Waldron, K. W. & Morris, V. J. Pectin - An emerging new bioactive food polysaccharide. *Trends Food Sci. Technol.* **24**, 64–73 (2012).

[70] Latarullo, M. B. G., Tavares, E. Q. P., Maldonado, G. P., Leite, D. C. C. & Buckeridge, M. S. Pectins, endopolygalacturonases, and bioenergy. *Front. Plant. Sci.* **7**, 1–7 (2016). 27703463 10.3389/fpls.2016.01401 PMC5028389

[71] Lionetti, V. et al. Engineering the cell wall by reducing de-methyl-esterified homogalacturonan improves saccharification of plant tissues for bioconversion. *Proc. Natl. Acad. Sci. U S A*. **107**, 616–621 (2010). 20080727 10.1073/pnas.0907549107 PMC2818903

[72] Wormit, A. & Usadel, B. The multifaceted role of pectin methylesterase inhibitors (PMEIs). *Int. J. Mol. Sci.* **19**, 1–19 (2018). 10.3390/ijms19102878 PMC6213510 30248977

[73] Moura, J. C. M. S., Bonine, C. A. V., de Oliveira Fernandes Viana, J., Dornelas, M. C. & Mazzafera, P. Abiotic and biotic stresses and changes in the lignin content and composition in plants. *J. Integr. Plant Biol.* **52**, 360–376 (2010). 20377698 10.1111/j.1744-7909.2010.00892.x

[74] Lee, M. et al. Lignin-based barrier restricts pathogens to the infection site and confers resistance in plants. *EMBO J.* **38**, 1–17 (2019). 10.15252/embj.2019101948 PMC6885736 31559647

[75] Oliveira, D. M. et al. Cell wall remodeling under salt stress: insights into changes in polysaccharides, feruloylation, lignification, and phenolic metabolism in maize. *Plant. Cell. Environ.* **43**, 2172–2191 (2020). 32441772 10.1111/pce.13805

[76] Dabravolski, S. A. & Isayenkov, S. V. The regulation of plant cell wall organisation under salt stress. *Front. Plant. Sci.* **14**, 1–16 (2023). 10.3389/fpls.2023.1118313 PMC10036381 36968390

[77] Chun, H. J. et al. Lignin biosynthesis genes play critical roles in the adaptation of Arabidopsis plants to high-salt stress. *Plant. Signal. Behav.* **14**, 1–4 (2019). 10.1080/15592324.2019.1625697 PMC6619940 31156026

[78] Cabane, M., Afif, D. & Hawkins, S. *Lignins and Abiotic Stresses*. *Advances in Botanical Research* vol. 61Elsevier Ltd., (2012).

[79] Liu, J., Zhang, W., Long, S. & Zhao, C. Maintenance of cell wall integrity under high salinity. *Int. J. Mol. Sci.* **22**, 1–19 (2021). 10.3390/ijms22063260 PMC8004791 33806816

[80] Yuan, L. et al. A glutathione S-transferase regulates lignin biosynthesis and enhances salt tolerance in tomato. *Plant. Physiol.* **196**, 2989–3006 (2024). 39324634 10.1093/plphys/kiae504

[81] Cesarino, I. Structural features and regulation of lignin deposited upon biotic and abiotic stresses. *Curr. Opin. Biotechnol.* **56**, 209–214 (2019). 30684783 10.1016/j.copbio.2018.12.012

[82] Yadav, S. & Chattopadhyay, D. Lignin: the Building block of defense responses to stress in plants. *J. Plant. Growth Regul.* **42**, 6652–6666 (2023).

[83] Li, X., Weng, J. K. & Chapple, C. Improvement of biomass through lignin modification. *Plant. J.* **54**, 569–581 (2008). 18476864 10.1111/j.1365-313X.2008.03457.x

[84] Skyba, O., Douglas, C. J. & Mansfield, S. D. Syringyl-Rich lignin renders poplars more resistant to degradation by wood decay fungi. *Appl. Environ. Microbiol.* **79**, 2560–2571 (2013). 23396333 10.1128/AEM.03182-12 PMC3623167

[85] Sadeghifar, H. & Ragauskas, A. J. Lignin as a natural antioxidant: chemistry and applications. *Macromol* **5**, 1–16 (2025).

[86] Rossi, L. et al. Salt stress induces differential regulation of the phenylpropanoid pathway in Olea Europaea cultivars Frantoio (salt-tolerant) and leccino (salt-sensitive). *J. Plant. Physiol.* **204**, 8–15 (2016). 27497740 10.1016/j.jplph.2016.07.014

[87] Karagoz, P. et al. Pharmaceutical applications of lignin-derived chemicals and lignin-based materials: linking lignin source and processing with clinical indication. *Biomass Convers. Biorefinery*. **14**, 26553–26574 (2024). 10.1007/s13399-023-03745-5 PMC11525408 39493283

[88] Ralph, J., Lapierre, C. & Boerjan, W. Lignin structure and its engineering. *Curr. Opin. Biotechnol.* **56**, 240–249 (2019). 30921563 10.1016/j.copbio.2019.02.019

[89] Ragauskas, A. J. et al. Lignin valorization: improving lignin processing in the biorefinery. *Science (80-)* 344, (2014). 10.1126/science.1246843 24833396

[90] Slama, I., Abdelly, C., Bouchereau, A., Flowers, T. & Savouré, A. Diversity, distribution and roles of osmoprotective compounds accumulated in halophytes under abiotic stress. *Ann. Bot.* **115**, 433–447 (2015). 25564467 10.1093/aob/mcu239 PMC4332610

[91] Parida, A. K. & Das, A. B. Salt tolerance and salinity effects on plants: A review. *Ecotoxicol. Environ. Saf.* **60**, 324–349 (2005). 15590011 10.1016/j.ecoenv.2004.06.010

[92] Shabala, S. Learning from halophytes: physiological basis and strategies to improve abiotic stress tolerance in crops. *Ann. Bot.* **112**, 1209–1221 (2013). 24085482 10.1093/aob/mct205 PMC3806534

[93] Thompson, D. S. & Islam, A. Plant cell wall hydration and plant physiology: an exploration of the consequences of direct effects of water deficit on the plant cell wall. *MDPI Plants* **10**, (2021). 10.3390/plants10071263 PMC8309141 34206199

[94] Cárdenas-Pérez, S. et al. Microstructure of Salicornia Bigelovii Stems under Photonic and Electron Microscopy. in *Microscopy and Microanalysis* vol. 26. (Cambridge University Press, 2020). (2020).

[95] Lu, C. et al. The dynamic remodeling of plant cell wall in response to heat stress. *Genes (Basel)*. **16**, 1–15 (2025). 10.3390/genes16060628 PMC12191967 40565520

[96] Ventura, Y., Eshel, A., Pasternak, D. & Sagi, M. The development of halophyte-based agriculture: past and present. *Ann. Bot.* **115**, 529–540 (2015). 25122652 10.1093/aob/mcu173 PMC4332600

