# Supplementary material for: Cell wall remodeling and polarized light analysis reveal ecotype-specific strategies in Salicornia europaea L. with biotechnological applications
Source: Sci Rep. 2025 Dec 8;16:964. doi: 10.1038/s41598-025-30480-w (PMC12783736; doi:10.1038/s41598-025-30480-w)
Supplement: Supplementary file 1 — Supplementary Material 1 [file 41598_2025_30480_MOESM1_ESM.docx]

Supplementary Information

**Table S1**. Cell-wall stiffness (Young’s modulus, E), fresh weight (FW) and dry weight (DW) biomass of Salicornia europaea populations Ciechocinek and Inowrocław grown under 0, 200, 400, and 1000 mM NaCl. Values are mean ± SD (n = 3); different letters indicate significant differences within NaCl treatments , * indicate significant differences among populations (*p* < 0.05) according to two-way ANOVA and Tukey HSD. Data for Inowrocław *E* and biomass are from Cárdenas-Pérez et al. (2024a,b)

| NaCl (mM) | Cell wall stiffness Ciechocinek, *E* (MPa) | Cell wall stiffness Inowrocław, *E* (MPa) ^1^ | Biomass FW Ciechocinek (g) | Biomass FW Inowrocław (g) ^2^ | Biomass DW Ciechocinek (g)^2^ | Biomass DW Inowrocław (g) ^2^ |
| --- | --- | --- | --- | --- | --- | --- |
| 0 | 1.790 ± 0.22^a^* | 0.518 ± 0.03^a^ | 3.74 ± 0.99^a^ | 3.44 ± 1.25^c^ | 0.37 ± 0.09^c^ | 0.40 ± 0.14^c^ |
| 200 | 1.278 ± 0.10^b^* | 0.278 ± .008^b^ | 9.73 ± 6.34^b^* | 5.85 ± 2.14^b^ | 0.84 ± 0.67^a^* | 0.56 ± 0.19^b^ |
| 400 | 1.304 ± 0.09^b^* | 0.102 ± 0.001^c^ | 10.10 ± 2.46^c^* | 15.74 ± 8.68^a^ | 0.62 ± 0.09^b^* | 1.14 ± 0.60^a^ |
| 1000 | 0.357 ± 0.008^c^* | 0.039 ± 0.004^d^ | 0.5 ± 0.27^d^* | 2.18 ± 0.403^d^ | 0.05 ± 0.026^d^* | 0.20 ± 0.04^d^ |

1. Cárdenas Pérez, S. *et al.* Salinity-driven changes in Salicornia cell wall nanomechanics and lignin composition. *Environ. Exp. Bot.* **218**, 105606 (2024).

2. Cárdenas Pérez, S., Grigore, M. N. & Piernik, A. Prediction of Salicornia europaea L. biomass using a computer vision system to distinguish different salt-tolerant populations. *BMC Plant Biol.* **24**, (2024).

**Table S2**. Pearson correlation matrix among cell-wall nanomechanical properties (*E* stiffness), polymer composition (pectin fractions, cellulose, lignin monomers and ratios), S: syringaldehyde + syringic acid, G: vanillin + vanillic acid, H: hydroxybenzaldehyde + *p*-hydroxybenzoic acid and total lignin yield, fresh weight (FW), polarized brightness area (Polz b. area) and polarized brightness intensity (Polz b. intensity) in *S. europaea* under four salinity treatments 0,200, 400 and 1000 mM NaCl.

| **Variables** | ***E* stiffness** | **Pectin HM-HG** | **Pectin LM-HG** | **Cellulose** | **S/G** | **H/G** | **S** | **G** | **H** | **Lignin-Total yield** | **FW** | **Polz. brigth area** | **Polz. intensity** |
| --- | --- | --- | --- | --- | --- | --- | --- | --- | --- | --- | --- | --- | --- |
| ***E* stiffness** | **1** | 0.556 | 0.579 | **0.950** | 0.239 | -0.002 | -0.080 | -0.602 | -0.889 | -0.349 | 0.477 | -0.353 | -0.853 |
| **Pectin HM-HG** | 0.556 | **1** | 0.431 | 0.708 | 0.907 | -0.825 | 0.744 | 0.275 | -0.755 | 0.535 | 0.897 | 0.386 | -0.166 |
| **Pectin LM-HG** | 0.579 | 0.431 | **1** | 0.759 | 0.039 | -0.024 | -0.167 | -0.506 | -0.825 | -0.354 | 0.022 | -0.612 | -0.740 |
| **Cellulose** | **0.950** | 0.708 | 0.759 | **1** | 0.367 | -0.186 | 0.061 | -0.484 | **-0.987** | -0.216 | 0.519 | -0.323 | -0.813 |
| **S/G** | 0.239 | 0.907 | 0.039 | 0.367 | **1** | **-0.960** | 0.948 | 0.626 | -0.412 | 0.824 | 0.944 | 0.738 | 0.242 |
| **H/G** | -0.002 | -0.825 | -0.024 | -0.186 | **-0.960** | **1** | **-0.981** | -0.768 | 0.268 | -0.912 | -0.813 | -0.775 | -0.415 |
| **S** | -0.080 | 0.744 | -0.167 | 0.061 | 0.948 | **-0.981** | **1** | 0.840 | -0.125 | **0.961** | 0.818 | 0.882 | 0.532 |
| **G** | -0.602 | 0.275 | -0.506 | -0.484 | 0.626 | -0.768 | 0.840 | **1** | 0.411 | **0.958** | 0.410 | 0.922 | 0.902 |
| **H** | -0.889 | -0.755 | -0.825 | **-0.987** | -0.412 | 0.268 | -0.125 | 0.411 | **1** | 0.146 | -0.516 | 0.302 | 0.764 |
| **Lignin-Total y.** | -0.349 | 0.535 | -0.354 | -0.216 | 0.824 | -0.912 | **0.961** | **0.958** | 0.146 | **1** | 0.646 | 0.943 | 0.745 |
| **FW** | 0.477 | 0.897 | 0.022 | 0.519 | 0.944 | -0.813 | 0.818 | 0.410 | -0.516 | 0.646 | **1** | 0.637 | 0.036 |
| **Polz. b. area** | -0.353 | 0.386 | -0.612 | -0.323 | 0.738 | -0.775 | 0.882 | 0.922 | 0.302 | 0.943 | 0.637 | **1** | 0.788 |
| **Polz. intensity** | -0.853 | -0.166 | -0.740 | -0.813 | 0.242 | -0.415 | 0.532 | 0.902 | 0.764 | 0.745 | 0.036 | 0.788 | **1** |
| *Values in bold are different from 0 with a significance level alpha=0.05* | | | | | | |  |  |  |  |  |  |  |
